# Reactions of nitroxides 15. Cinnamates bearing a nitroxyl moiety synthesized using a Mizoroki–Heck cross-coupling reaction

**DOI:** 10.3762/bjoc.11.130

**Published:** 2015-07-13

**Authors:** Jerzy Zakrzewski, Bogumiła Huras

**Affiliations:** 1 Institute of Industrial Organic Chemistry, Annopol 6, 03-236 Warsaw, Poland

**Keywords:** 4-acryloyloxy-2,2,6,6-tetramethylpiperidine-1-oxyl, cinnamates, Mizoroki–Heck cross-coupling reaction, nitroxides

## Abstract

Cinnamic acid derivatives bearing a nitroxyl moiety (2,2,6,6-tetramethyl-1-oxyl-4-piperidyl 3-*E*-aryl acrylates) were synthesized in 30–100% yield using a Mizoroki–Heck cross-coupling reaction between 4-acryloyloxy-2,2,6,6-tetramethylpiperidine-1-oxyl and iodobenzene derivatives in the presence of palladium(II) acetate coordinated with a tri(*o*-tolyl)phosphine ligand immobilized in a polyurea matrix.

## Introduction

Cinnamic acid derivatives are known as biologically active compounds. Cinnamic acid and its hydroxy derivatives bearing a phenolic moiety such as coumaric, caffeic, ferulic, sinapinic, and chlorogenic acids [2–6], simple cinnamic acid derivatives (esters, amides, etc.), and prenylated cinnamates [4], have been proved to be effective as antioxidants [2,7], radical scavengers [2], antimicrobials [2,7–8], antibacterials [2], antivirals [2,7], and fungicides [2,7–8]. Cinnamic derivatives have also been recognized as active agents against tuberculosis and malaria [3,7], cardiovascular diseases [3], and cancer [4]. Cinnamates show depigmenting [4], antidiabetic, antihyperglycemic, anticholesterolemic, anti-inflammatory, hepatoprotective, CNS depressant, anxiolytic, and cytotoxic activity [7]. Cinnamate esters have been used as effective UV filters (especially in UVB region, 280–320 nm) in cosmetics [7,9], and as fragrance materials [7]. Fragrance material reviews on cinnamic acid derivatives were extensively described in *Food and Chemical Toxicology* (especially in 2007) and in *Food and Cosmetics Toxicology* (in the 70s). The most recent reviews in this series were published in 2011 [10–11].

Cinnamic acid derivatives can be synthesized using Perkin, Knoevenagel, Claisen [7], and Wittig [12] condensation reactions. Since the discovery of catalytic coupling reactions, cinnamic derivatives have also been obtained using the Mizoroki–Heck cross-coupling reaction between aryl halides and an olefin [7,13–17]. Because cinnamates themselves are also olefins, they can serve as cinnamic substrates to synthesize more complex cinnamates [18–19]. Cinnamic acid derivatives are also formed when saturated aliphatic acids (instead of unsaturated ones as acrylates) are reacted with simple aromatic compounds (as benzene) in the presence of palladium(II) chloride [20]. Due to the important biological activity of cinnamates, the incorporation of a spin label moiety, particularly a nitroxyl fragment, is interesting, because it allows for the study of cinnamates using spin labeling methods.

To accomplish the synthesis of cinnamates bearing a nitroxyl moiety, we applied a Mizoroki–Heck cross-coupling reaction, using 4-acryloyloxy-2,2,6,6-tetramethylpiperidine-1-oxyl (**3**, an olefin component bearing a nitroxyl moiety), recently applied in the Morita–Baylis–Hillmann reaction [21]. The use of nitroxides in cross-coupling reactions is described only in a limited number of papers [22–32]. To the best of our knowledge, there are no systematic studies on the use of nitroxides in the Mizoroki–Heck cross-coupling reaction.

Despite the observation that an unpaired electron in nitroxides does not participate in organic reactions has been well known since the beginning of the nitroxide progress in 60's [33] the reactions of nitroxides involving an unpaired electron are also recognized (e.g., reductions, acidic medium, carbene addition, etc.). Because we would like to check the possibility of performing the Mizoroki–Heck cross-coupling reaction with nitroxides, herein the synthesis of cinnamates bearing a nitroxyl moiety is presented using aryl iodides as exemplary test compounds.

## Results and Disscusion

4-Acryloyloxy-2,2,6,6-tetramethylpiperidine-1-oxyl (**3**) was obtained in 90–95% yield by the reaction of acryloyl chloride (**2**) with 2,2,6,6-tetramethyl-4-piperidinol-1-oxyl (**1**) in the presence of triethylamine [21,34–36].

The couplings of 4-acryloyloxy-2,2,6,6-tetramethylpiperidine-1-oxyl (**3**) with iodobenzene (**4a**) and 4-methyliodobenzene (**4b**) were used as the test reactions to check the effectiveness of various palladium catalyst systems.

The use of Pd(OAc)_2_/Ph_3_P/Bu_3_N [37] resulted in a low yield of the target products. No products were obtained when other catalyst systems: Pd[PPh_3_]_4_ [38], Pd(CF_3_COO)_2_/tri(2-furylphosphine) and Pd(acetylacetonate)_2_/tri(2-furylphosphine) [39] were tested. As a rule, unreacted **3** was identified, and always isolated. A number of unidentified products were detected by means of TLC.

**Scheme 1:**
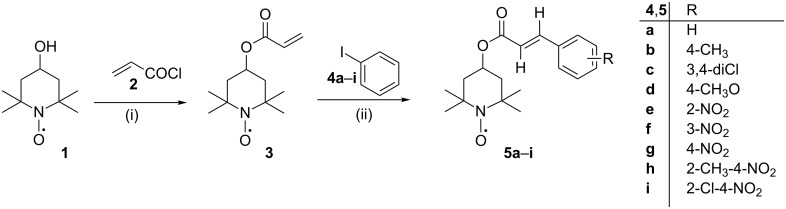
Cinnamates bearing a nitroxyl moiety **5a**–**i** from 4-acryloyloxy-2,2,6,6-tetramethylpiperidine-1-oxyl (**3**) and iodobenzene derivatives; (i) CH_2_=CH–COCl (**2**), NEt_3_, benzene, 90–95%; (ii) R–Ar–I **4a**–**i**, PdEnCat TOTP30, Bu_4_N^+^AcO^−^, toluene, 80–100 °C, 30–100%.

Finally, the target cinnamates bearing a nitroxyl moiety (**5a**–**i**) were obtained using the coupling of 4-acryloyloxy-2,2,6,6-tetramethylpiperidine-1-oxyl (**3**) with a series of iodobenzene derivatives (**4a**–**i**) in the presence of palladium(II) acetate coordinated with tri(*o*-tolyl)phosphine ligand immobilized in a polyurea matrix (commercially available as PdEnCatTOTP30™) [40–45] (Scheme 1, Table 1).

**Table 1:** Cinnamates bearing a nitroxyl moiety (**5a**–**i**).

Compound	R	Reaction temperature [°C]	Reaction time [h]	Yield [%]	mp [°C]

**5a**	H	80	22	42.4	97–98
**5b**	4-CH_3_	100	2–20	30.4	oil
**5c**	3,4-diCl	80	25	62.7	118–120
**5d**	4-CH_3_O	85	24	75.8	70–74
**5e**	2-NO_2_	90	20	76.4	107–110
**5f**	3-NO_2_	80–90	20	99.9	121–123
**5g**	4-NO_2_	80–85	23	55.3	148–149
**5h**	2-CH_3_-4-NO_2_	80–85	27	88.3	109–112
**5i**	2-Cl-4-NO_2_	80–85	27	56.2	107–110

The best results were obtained when an electron-withdrawing substituent NO_2_ was present in the benzene ring. The reactions of iodobenzene bearing 4-F, 4-CF_3_, 2,4-diNO_2_ substituents were unsuccessful. In the case of the reaction of **4b** leading to the cinnamate **5b**, the results were unrepeatable (the times and yields of the reactions). Unidentified impurities together with the product **5b** were observed (^13^C NMR).

No products in the case of 4-F, 4-CF_3_, 2,4-diNO_2_ substituents and unrepeatability in the case of cinnamate **5b** can be caused by means of a type of a heterogeneous catalyst which is immobilized on a solid support. In addition to the widely highlighted unquestionable advantages of immobilized, heterogeneous catalysts (easy separation from the reaction mixture and the possibility of re-use), such catalysts are rigidly anchored on a carrier and may cause hindered interaction with substrates. Some of the catalytic sites may be buried within the polymer matrix and cannot participate in the reactions [46]. This disadvantage may cause various unwanted effects. As examples, no clear relationship between the structures of the aryl iodides and the yields were observed, as described in [47], or a catalyst that accepts only electron-rich aryl iodides [48].

The structure of the synthesized cinnamates bearing a nitroxyl moiety were confirmed using EIMS, ESIMS, HREIMS, HRESIMS, ^1^H NMR, ^13^C NMR, and IR spectra (Experimental part and Supporting Information File 1). Recording of the NMR spectra of the nitroxides required removing of the unpaired electron. This was achieved by adding a drop of a reducing agent – in fact the spectra of the corresponding hydroxylamines were recorded. In this research phenylhydrazine has been used [49–50], however, sometimes using of hydrazobenzene [51–52] or pentafluorophenyl hydrazine [35] as reducing agents have been reported, as well.

The *E* geometry of **5a**–**i** was confirmed by ^1^H NMR spectroscopy. The values of the coupling constants of the doublets visible in the ^1^H NMR spectra of **5a**–**i** belonging to the double bond of **5a**–**i** remain in the range 15.5–16.0 Hz. These values confirm the *E* geometry of **5a**–**i**. In Table 2 the shifts and the coupling constants of the double bond are presented.

**Table 2:** Chemical shifts and coupling constants of the hydrogen atoms belonging to the double bond of **5a**–**i**: OOC–CH^(B)^=CH^(D)^–C_6_H_4(3)_–R.

**5**	R	δ H^(B)^ [ppm]	δ H^(D)^ [ppm]	*J*_DB_ [Hz]

**a**	H	6.41	7.67	16.0
**b**	4-CH_3_	6.35	7.64	15.8
**c**	3,4-diCl	6.38	7.40	15.5
**d**	OCH_3_	6.28	7.62	16.0
**e**	2-NO_2_	6.33	8.10	16.0
**f**	3-NO_2_	6.53	7.69	16.0
**g**	4-NO_2_	6.52	7.32	16.0
**h**	2-CH_3_-4-NO_2_	6.42	7.90	15.9
**i**	2-Cl-4-NO_2_	6.51	8.03	16.0

**Figure 1:**
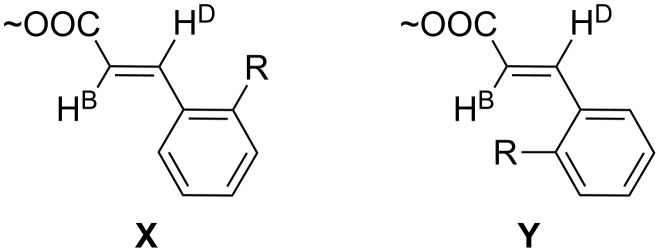
Conformations of the substituent in 2-position of the cinnamates **5e**, **5h**, **5i**.

The substituent in 2-position of the cinnamates **5e**, **5h**, **5i** causes the noticeable shift of H^D^ hydrogen to the lower field. This observation suggests that conformation X, where the substituent in 2-position and the H^D^ atom are placed close to each other, is more populated than conformation Y (Figure 1).

For cinnamate **5f** (3-NO_2_), the direct measurement of the chemical shift of the hydrogen atom H^D^ (OOC-CH^(B)^=C**H^(D)^**-C_6_H_4_-NO_2_^(m)^) was not possible, due to the presence of more than one equidistant (16 Hz) peak close to 7.5 ppm. Irradiation of H^B^ (δ 6.53 ppm) allowed to distinguish the signal of H^D^ at 7.69 ppm.

A molecular peak was present in all mass spectra of the synthesized products **5a**–**5i**. Except in the case of **5c**,**i** it was clearly visible (5–25%). In all of the mass spectra, the abundant peak of the cinnamyl acyl cation ArCH=CHCO^+^ was present. This was a base peak in the case of **5a,d**.

**Scheme 2:**
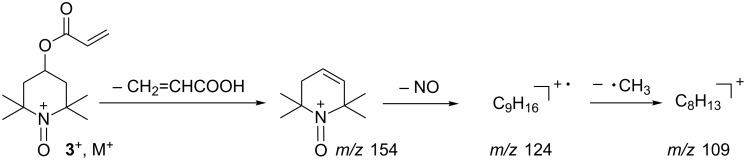
The formation of the fragment ions at *m*/*z* 154, 124, 109.

Characteristic signals for 4-*XO*-substituted 2,2,6,6-tetramethylpiperidine-1-oxyl moiety (TEMPOL, TEMPOL esters, etc.) were observed at *m*/*z* = 154, 124, 109 [53–54]. The signals at *m*/*z* 124, and 109 are abundant. The signal at *m*/*z* 154 was assigned to the structure, resulting from elimination of a XOH from the position 4 and 3 of the piperidine ring. The subsequent loss of a NO group (M = 30) and a CH_3_ group (M = 15), respectively, generates ions at *m*/*z* 124 and 109 (Scheme 2). The peaks at *m*/*z* 154, 124 and 109 were thoroughly analyzed for the acrylate **3**, as an example.

Proposed elemental composition of the fragmentation ions at *m*/*z* 154, 124, 109 for the acrylate **3** were confirmed by the measurement of their exact mass:

- *m*/*z* 154 (calculated C_9_H_16_NO: 154.12319, found: 154.12390),
- *m*/*z* 124 (calculated for C_9_H_16_: 124.12520, found: 124.12515),
- *m*/*z* 109 (calculated for C_8_H_13_: 109.10173, found: 109.10079).

The signal at *m*/*z* 124 is the base one in the case of **5b**,**e**,**g**,**h**, and that at *m*/*z* 109 in the case of **5c**,**f**,**i**. The *m*/*z* values and relative intensities of the discussed signals are collected in Table 3.

**Table 3:** The relative intensities of the molecular, cinnamic acyl, *m*/*z* 154, *m*/*z* 124, and *m*/*z* 109 ions.

**5**	M [*m*/*z* (int.%)]	ArCH=CHCO [*m*/*z* (int.%)]	*m*/*z* 154 int. %	*m*/*z* 124 int. %	*m*/*z* 109 int. %

**a**	302 (10)	131 (100, C_6_H_5_CH=CHCO)	27	89	69
**b**	316 (24)	145 (91, 4-CH_3_C_6_H_4_CH=CHCO)	46	100	75
**c**	370 (2)	199 (64, 3,4-Cl_2_CH_3_C_6_H_3_CH=CHCO)	16	88	100
**d**	332 (19)	161 (100, 4-CH_3_OC_6_H_4_CH=CHCO)	20	61	44
**e**	347 (23)	176 (63, 2-NO_2_C_6_H_4_CH=CHCO)	25	100	87
**f**	347 (5)	176 (85, 3-NO_2_C_6_H_4_CH=CHCO)	13	83	100
**g**	347 (19)	176 (89, 4-NO_2_C_6_H_4_CH=CHCO)	19	100	90
**h**	361 (15)	190 (74, 2-CH_3_-4-NO_2_C_6_H_4_CH=CHCO)	29	100	82
**i**	381 (2)	210 (41, 2-Cl-4-NO_2_C_6_H_4_CH=CHCO)	15	89	100

In conclusion, we showed that 4-acryloyloxy-2,2,6,6-tetramethylpiperidine-1-oxyl (**3**) can be used as starting material in the synthesis of new cinnamates containing a nitroxyl group in a Mizoroki–Heck cross-coupling reaction.

## Experimental

### General

The protocols for the synthesis of 4-acryloyloxy-2,2,6,6-tetramethylpiperidine-1-oxyl (**3**) and its precursor 2,2,6,6-tetramethyl-4-hydroxypiperidin-1-oxyl (TEMPOL, **1**) were done according to [21]. The identity of **3** was additionally confirmed by ^1^H and ^13^C NMR performed in the presence of PhNHNH_2_. ^1^H NMR (200 MHz, δ, CDCl_3_, TMS, in the presence of PhNHNH_2_) 1.23 (s, 6H, (C*H*_3_)(CH_3_)CN(OH)C(CH_3_)(C*H*_3_), 1.24 (s, 6H, (CH_3_)(C*H*_3_)CN(OH)C(C*H***_3_**)(CH_3_)), 1.65 (t, *J* = 11.8 Hz, 2H, *H*HC-CH(O-)-CH*H*), 1.96 (ddt, *J* = 12.8 Hz, *J* = 4.2 Hz, *J* = 1.5 Hz, 2H, H*H*C-CH(O-)-C*H*H), 5.13 (tt, *J* = 11.2 Hz, *J* = 4.3 Hz, 1H, C*H*-OC(=O)), 5.81 (dd, *J* = 10.3 Hz, *J* = 1.6 Hz, 1H, C*H*H=CH-COO *(cis)*), 6.09 (dd, *J* = 17.2 Hz, *J* = 10.3 Hz, 1H, CH_2_=C*H*-COO), 6.39 (dd, *J* = 17.2 Hz, *J* = 1.6 Hz, 1H, CH*H*=CH-COO (*trans*)); ^13^C NMR (50 MHz, δ, CDCl_3_, TMS, in the presence of PhNHNH_2_) 20.79 (2×CH_3_), 31.86 (2×CH_3_), 43.92 (2×CH_2_), 59.89 (2×*C*(CH_3_)_2_), 66.99 (CH-O-), 128.94 (CO*C*H=CH_2_), 130.84 (COCH=*C*H_2_), 165.95 (C=O).

PdEnCatTOTP30 was purchased from Aldrich. Iodobenzene derivatives and catalysts were purchased from Aldrich, Alfa-Aesar and Fluorochem. The Pd(PPh_3_)_4_ was synthesized according to the known procedure [38]. The experiments were performed in a 25–50 mL round-bottom two necked flask, equipped with a magnetic stirrer and a reflux condenser under anhydrous argon atmosphere (inlet through the top of the condenser equipped with a short drying column packed with drying silica, outlet by a needle placed in a septum in a side neck of the flask). Most of the products were obtained as red solids. TLC was performed on silica gel Merck aluminium roll 5562 or aluminium sheets 5554. Column chromatography was performed using Merck 1.09385.1000 or Zeochem 60 hyd 40–63 μm silica gel (0.040–0.063 mm, 230–400 mesh), respectively. TLC visualisation: UV 254 nm light and/or iodine vapours. MS (EI, 70 eV, *m*/*z*, int. (%)) data were recorded using AMD 604 and Agilent Technologies 5975 B mass spectrometers. HRMS (EI) data were recorded using an AMD 604 mass spectrometer. MS and HRMS (ESI, positive ion, CH_3_OH as a solvent) were recorded using a Micromass LCT apparatus. IR (cm^−1^) data were recorded using a FTIR Jasco 420 spectrometer. ^1^H and ^13^C NMR data were collected using a Varian UNITYplus 200 spectrometer. NMR spectra were performed with a drop of phenylhydrazine (in fact the spectra of corresponding hydroxylamines were recorded) [49].

### Cinnamates **5a–i**; general procedure using PdEnCatTOTP30 as a catalyst

4-Acryloyloxy-2,2,6,6-tetramethylpiperidin-1-oxyl (**3**, 0.113 g, 0.5 mmol), Aryl iodide (**4a**–**i**, 0.5 mmol), Bu_4_N^+^AcO^−^ (hygroscopic) (0.3 g), PdEnCatTOTP30 (0.0625 g, 0.025 mol, 5 mol %), and toluene (2 mL) were placed in a flask, stirred and heated at 80–100 °C for 20–27 h under argon. The particular temperatures and times of the reactions are summarized in Table 1. After approximately 6 h, a second portion of a catalyst (0.0625 g, 0.025 mol, 5 mol %) was added. The progress of the reaction was monitored by TLC (silica, hexane/ethyl acetate 9:1). Upon completion of the reaction, the solids were filtered off, the dark filtrate was concentrated under reduced pressure and subjected to column chromatography (hexane/ethyl acetate 9:1, benzene/ethyl acetate 95:5, benzene/methanol 95:5 as possible mobile phases). The red zone was collected, the eluate was evaporated under reduced pressure to yield red oils (solidified in a refrigerator), or directly red crystals of **5a**–**i**.

### 2,2,6,6-Tetramethyl-1-oxyl-4-piperidyl 3-*E-*phenylacrylate (**5a**)

42.4%; mp 97–98 °C; MS (EI, 70 eV, *m*/*z*, int [%]) 302 (10, M^+^), 272 (7), 207 (19), 179 (6), 178 (10), 154 (27), 140 (20), 139 (14), 131 (100, ArCH=CHCO), 124 (89), 109 (69), 103 (41), 82 (15), 81 (12), 77 (21), 69 (13), 68 (9), 67 (9), 55 (10), 41 (18); HRMS (EI, 70 eV, *m/z*, int [%]): calcd for C_18_H_24_NO_3_: 302.1756, found: 302.1750; ^1^H NMR (200 MHz, δ, CDCl_3_, TMS, PhNHNH_2_) 1.26 (s, 6H, (C*H*_3_)(CH_3_)CN(OH)C(CH_3_)(C*H*_3_), 1.28 (s, 6H, (CH_3_)(C*H*_3_)CN(OH)C(C*H*_3_)(CH_3_)), 1.74 (t, *J* = 12.0 Hz, 2H, *H*HC-CH(O-)-CH*H*), 2.02 (ddd, *J* = 12.8 Hz, *J* = 4.4 Hz, *J* = 1.2 Hz, 2H, H*H*C-CH(O-)-C*H*H), 5.20 (tt, *J* = 11.2 Hz, *J* = 4.3 Hz, 1H, C*H*-OC(=O)), 6.41 (d, *J* = 16.0 Hz, 1H, Ar-CH=C*H*-COO), 7.67 (d, *J* = 16.0 Hz, 1H, Ar-C*H*=CH-COO); ^13^C NMR (50 MHz, δ, CDCl_3_, TMS, PhNHNH_2_) 20.87 (2×CH_3_), 31.67 (2×CH_3_), 43.87 (2×CH_2_), 60.46 (2×*C*(CH_3_)_2_), 66.76 (CH-O-), 118.51 (CH), 128.30 (CH), 129.10 (CH), 130.52 (CH), 134.57 (C), 145.00 (CH), 166.68 (C=O); IR (cm^−1^, KBr) 2976, 2937, 1712, 1639, 1450, 1308, 1168, 1008, 978, 859, 765, 709, 685.

### 2,2,6,6-Tetramethyl-1-oxyl-4-piperidyl 3-*E*-(4-methylphenyl)acrylate (**5b**)

30.4%, oil; MS (EI, 70 eV, *m*/*z*, int [%]) 316 (24, M^+^), 286 (15), 230 (6), 163 (13), 162 (21), 154 (46), 145 (91, ArCH=CHCO), 140 (62), 139 (27), 124 (100), 117 (43), 115 (49), 109 (75), 98 (14), 91 (32), 82 (37), 81 (22), 69 (31), 68 (17), 67 (18), 65 (10), 60 (8), 59 (9), 58 (11), 57 (13), 56 (19), 55 (25), 43 (35), 41 (39); MS (ESI, *m*/*z*, int [%]): 340 (15, M + 23 + H), 318 (M + 2H); HRMS (EI, 70 eV, *m*/*z*, int [%]): calcd for C_19_H_26_NO_3_: 316.1913, found: 316.1926; ^1^H NMR (200 MHz, δ, CDCl_3_, TMS, PhNHNH_2_) 1.85 (t, *J* = 12.1 Hz, 2H, *H*HC-CH(O-)-CH*H*), 2.02–2.17 (m, 2H, H*H*C-CH(O-)-C*H*H), 2.37 (s, 3H, CH_3_), 5.23 (tt, *J* = 11.1 Hz, *J* = 4.4 Hz, 1H, C*H*-OC(=O)), 6.35 (d, *J* = 15.8 Hz, 1H, Ar-CH=C*H*-COO), 7.64 (d, *J* = 15.8 Hz, 1H, Ar-C*H*=CH-COO); ^13^C NMR (50 MHz, δ, CDCl_3_, TMS, PhNHNH_2_): 21.35 (impurity), 21.69 (2×CH_3_), 23.04 (Ar*C*H_3_), 29.84 (2×CH_3_), 43.01 (2×CH_2_), 62.60 (2×*C*(CH_3_)_2_), 65.67 (CH-O-), 115.32 (impurity), 117.03 (CH), 128.34 (CH), 129.86 (CH), 141.12 (C), 145.37 (CH), 166.73 (C=O), 179.47 (impurity); IR (cm^−1^, film): 2976, 1711, 1635, 1166, 815.

### 2,2,6,6-Tetramethyl-1-oxyl-4-piperidyl 3-*E*-(3,4-dichlorophenyl)acrylate (**5c**)

62.7%; pink crystals, mp 118–120 °C; MS (EI, 70 eV, *m/z*, int [%]) 370 (2, M^+^), 201 (40), 199 (64, ArCH=CHCO), 173 (12), 171 (19), 154 (16), 136 (42), 124 (88), 109 (100), 101 (8), 99 (11), 82 (16), 81 (19), 69 (18), 67 (21), 56 (17), 55 (25), 41 (65); MS (ESI, *m/z*, int [%]): 395 (20), 393 (90, M + Na), 304 (100); HRMS (ESI): calcd for C_18_H_22_NO_3_Cl_2_Na: 393.0874, found, 393.0890; ^1^H NMR (200 MHz, δ, CDCl_3_, TMS, PhNHNH_2_) 1.27 (s, 6H, (C*H*_3_)(CH_3_)CN(OH)C(CH_3_)(C*H*_3_)), 1.29 (s, 6H, (CH_3_)(C*H*_3_)CN(OH)C(C*H*_3_)(CH_3_)), 1.75 (t, *J* = 12.0 Hz, 2H, *H*HC-CH(O-)-CH*H*), 2.02 (dd, *J* = 12.8 Hz, *J* = 4.4 Hz, 2H, H*H*C-CH(O-)-C*H*H), 5.20 (tt, *J* = 11.3 Hz, *J* = 4.3 Hz, 1H, C*H*-OC(=O)), 6.38 (d, *J* = 15.5 Hz, 1H, Ar-CH=C*H*-COO), 7.40 (d, *J* = 15.5 Hz, 1H, Ar-C*H*=CH-COO); ^13^C NMR (50 MHz, δ, CDCl_3_, TMS, PhNHNH_2_) 20.87 (2×CH_3_), 31.54 (2×CH_3_), 43.72 (2×CH_2_), 60.70 (2×*C*(CH_3_)_2_), 67.04 (CH-O-), 117.10 (C), 120.36 (CH), 127.23 (CH), 129.85 (CH), 131.10 (CH), 133.45 (C), 134.59 (C), 142.24 (CH), 166.02 (C=O); IR (cm^−1^, KBr) 2975, 1706, 1638, 1474, 1319, 1267, 1173, 1150, 1206, 979, 822.

### 2,2,6,6-Tetramethyl-1-oxyl-4-piperidyl 3-*E*-(4-methoxyphenyl)acrylate (**5d**)

75.8%; mp 70–74 °C; MS (EI, 70 eV, *m*/*z*, int [%]) 332 (19, M^+^), 302 (6), 284 (2), 267 (5), 246 (4), 178 (32), 161 (100, ArCH=CHCO), 154 (20), 140 (8), 139 (9), 133 (17), 124 (61), 109 (44); MS (ESI, *m*/*z*, int [%]): 355 (100, M + Na); HRMS (ESI): calcd for C_19_H_26_NO_4_Na: 355.1760, found, 355.1753; ^1^H NMR (200 MHz, δ, CDCl_3_, TMS, PhNHNH_2_) 1.24 (s, 6H, (C*H*_3_)(CH_3_)CN(OH)C(CH_3_)(C*H*_3_)), 1.25 (s, 6H, (CH_3_)(C*H*_3_)CN(OH)C(C*H*_3_)(CH_3_)), 1.67 (t, *J* = 11.9 Hz, 2H, *H*HC-CH(O-)-CH*H*), 2.00 (ddd, *J* = 11.2 Hz, *J* = 4.4 Hz, *J* = 1.4 Hz, 2H, H*H*C-CH(O-)-C*H*H), 1.67 (t, *J* = 11.9 Hz, 2H, *H*HC-CHOCO-CH*H*), 2.00 (ddd, *J* = 1.4 Hz, *J* = 4.4 Hz, *J* = 11.2 Hz, 2H, H*H*C-CHOCO-C*H*H), 3.83 (s, 3H, OCH_3_), 5.18 (tt, *J* = 11.2 Hz, *J* = 4.4 Hz, 1H, C*H*-OC(=O)), 6.28 (d, *J* = 16.0 Hz, 1H, Ar-CH=C*H*-COO), 7.62 (d, *J* = 16.0 Hz, 1H, Ar-C*H*=CH-COO); ^13^C NMR (50 MHz, δ, CDCl_3_, TMS, PhNHNH_2_) 20.79 (2×CH_3_), 31.96 (2×CH_3_), 44.12 (2×CH_2_), 55.57 (OCH_3_), 59.90 (2×*C*(CH_3_)_2_), 66.68 (CH-O-), 114.51 (CH), 116.03 (CH), 127.33, 129.94 (CH), 144.60 (CH), 161.57, 167.02 (C=O); IR (cm^−1^, KBr) 1707, 1632, 1604, 1515, 1290, 1255, 1163, 984, 827.

### 2,2,6,6-Tetramethyl-1-oxyl-4-piperidyl 3-*E*-(2-nitrophenyl)acrylate (**5e**)

76.4%; mp 107–110 °C; MS (EI, 70 eV, *m*/*z*, int [%]) 347 (23, M^+^), 317 (5), 176 (63, ArCH=CHCO), 154 (25), 140 (11), 139 (17), 130 (61), 124 (100), 109 (87), 102 (21); MS (ESI, *m*/*z*, int [%]): 370 (100, M + Na); HRMS (ESI): calcd for C_18_H_23_N_2_O_5_Na: 370.1505, found, 370.1492; ^1^H NMR (200 MHz, δ, CDCl_3_, TMS, PhNHNH_2_) 1.24 (s, 6H, (C*H*_3_)(CH_3_)CN(OH)C(CH_3_)(C*H*_3_)), 1.25 (s, 6H, (CH_3_)(C*H*_3_)CN(OH)C(C*H*_3_)(CH_3_)), 1.69 (t, *J* = 11.8 Hz, 2H, *H*HC-CH(O-)-CH*H*), 2.01 (ddd, *J* = 12.0 Hz, *J* = 3.4 Hz, *J* = 1.3 Hz, 2H, H*H*C-CH(O-)-C*H*H), 1.69 (t, *J* = 11.8 Hz, 2H, *H*HC-CHOCO-CH*H*), 2.01 (dtd, *J* = 1.4 Hz, *J* = 3.4 Hz, *J* = 12.0 Hz, 2H, H*H*C-CHOCO-C*H*H), 5.20 (tt, *J* = 11.3 Hz, *J* = 4.3 Hz, 1H, C*H*-OC(=O)), 6.33 (d, *J* = 16.0 Hz, 1H, Ar-CH=C*H*-COO), 8.10 (d, *J* = 16.0 Hz, 1H, Ar-C*H*=CH-COO); ^13^C NMR (50 MHz, δ, CDCl_3_, TMS, PhNHNH_2_) 20.82 (2×CH_3_) 31.91 (2×CH_3_) 43.96 (2×CH_2_) 59.84 (2×*C*(CH_3_)_2_) 67.55 (CH-O) 123.60 (CH) 125.14 (CH) 129.34 (C) 130.49 (CH) 130.80 (C) 133.73 (CH) 140.27 (CH) 148.00 (CH) 165.51 (C=O); IR (cm^−1^, KBr) 2977, 1717, 1632, 1523, 1341, 1292, 1272, 1171, 1009, 980, 866, 789, 758.

### 2,2,6,6-Tetramethyl-1-oxyl-4-piperidyl 3-*E*-(3-nitrophenyl)acrylate (**5f**)

99.9%; mp 121–123 °C; MS (EI, 70 eV, *m*/*z*, int [%]) 347 (5, M^+^), 176 (85, ArCH=CHCO), 160 (6), 154 (13), 130 (17), 129 (17), 124 (83), 118 (12), 109 (100), 102 (73), 91 (13), 82 (17), 81 (22), 76 (21), 69 (18), 68 (12), 67 (25), 57 (14), 56 (18), 55 (27), 43 (19). 41 (62); MS (ESI, *m*/*z*, int [%]): 370 (100, M + Na); HRMS (ESI): calcd for C_18_H_23_N_2_O_5_Na: 370.1505, found, 370.1489; ^1^H NMR (200 MHz, δ, CDCl_3_, TMS, PhNHNH_2_), 1.249 (s, 6H, (C*H*_3_)(CH_3_)CN(OH)C(CH_3_)(C*H*_3_)), 1.254 (s, 6H, (CH_3_)(C*H*_3_)CN(OH)C(C*H*_3_)(CH_3_)), 1.69 (t, *J* = 12.0 Hz, 2H, *H*HC-CHOCO-CH*H*), 2.01 (ddd, *J* = 1.4 Hz, *J* = 4.4 Hz, *J* = 11.2 Hz, 2H, H*H*C-CHOCO-C*H*H), 5.21 (tt, *J* = 11.4 Hz, *J* = 4.4 Hz, 1H, CH-OC(=O)), 6.53 (d, *J* = 16.0 Hz, 1H, Ar-CH=C*H*-COO), 7.69 (d, *J* = 16.0 Hz, 1H, Ar-C*H*=CH-COO); ^13^C NMR (50 MHz, δ, CDCl_3_, TMS, PhNHNH_2_) 20.75 (2×CH_3_), 32.05 (2×CH_3_), 44.05 (2×CH_2_), 59.76 (2×*C*(CH_3_)_2_), 67.55 (CH-O-), 121.77 (CH), 122.63 (CH), 124.74 (CH), 130.16 (CH), 133.85 (CH), 136.32 (C), 142.00 (CH), 148.87 (C), 165.83 (C=O); IR (cm^−1^, KBr) 3080, 2971, 1709, 1642, 1526, 1358, 1330, 1191, 983, 813, 671.

### 2,2,6,6-Tetramethyl-1-oxyl-4-piperidyl 3-*E*-(4-nitrophenyl)acrylate (**5g**)

55.3%; mp 148–149 °C; MS (EI, 70 eV, *m*/*z*, int [%]) 347 (19, M^+^), 317 (6), 261 (3), 176 (89, ArCH=CHCO), 160 (7), 154 (19), 140 (14), 139 (15), 130 (24), 124 (100), 109 (90), 102 (29); HRMS (EI, 70 eV, *m*/*z*, int [%]): calcd for C_18_H_23_N_2_O_5_: 347.16070, found, 347.16009; ^1^H NMR (200 MHz, δ, CDCl_3_, TMS, PhNHNH_2_) 1.25 (s, 12H, 4×CH_3_), 1.68 (t, *J* = 12.0 Hz, 2H, *H*HC-CH(O-)-CH*H*), 2.00 (dd, *J* = 12.6 Hz, *J* = 4.0 Hz, 2H, H*H*C-CH(O-)-C*H*H), 1.68 (t, *J* = 12.0 Hz, 2H, *H*HC-CHOCO-CH*H*), 2.00 (dd, *J* = 4.0 Hz, *J* = 12.6 Hz, 2H, H*H*C-CHOCO-C*H*H), 5.21 (tt, *J* = 11.3 Hz, *J* = 4.4 Hz, 1H, C*H*-OC(=O)), 6.52 (dd, *J* = 16.0 Hz, *J* = 0.6 Hz, 1H, Ar-CH=C*H*-COO), 7.32 (dd, *J* = 16.0 Hz, *J* = 0.6 Hz, 1H, Ar-C*H*=CH-COO); ^13^C NMR (50 MHz, δ, CDCl_3_, TMS, PhNHNH_2_) 20.73 (2×CH_3_), 32.01 (2×CH_3_), 44.02 (2×CH_2_), 59.79 (2×*C*(CH_3_)_2_), 67.62 (CH-O-), 122.89 (CH), 124.37 (CH), 128.54 (C), 128.84 (CH), 140.69 (C), 141.92 (CH), 165.71 (C=O); IR (cm^−1^, KBr) 1705, 1638, 1519, 1344, 1177, 850.

### 2,2,6,6-Tetramethyl-1-oxyl-4-piperidyl 3-*E*-(2-methyl-4-nitrophenyl)acrylate (**5h**)

88.3%; mp 109–112 °C; MS (EI, 70 eV, *m*/*z*, int [%]) 361 (15, M^+^), 347 (4), 331 (7), 191 (32), 190 (74, ArCH=CHCO), 174 (11), 161 (6), 160 (10), 154 (29), 144 (37), 140 (57), 139 (26), 124 (100), 116 (47), 115 (60), 109 (82), 98 (12), 95 (11), 89 (10), 82 (36), 81 (30), 69 (33), 68 (20), 67 (27), 58 (9), 57 (12), 56 (21), 55 (30), 43 (15), 42 (12), 41 (45); HRMS (EI, 70 eV, *m*/*z*, int [%]): calcd for C_19_H_25_N_2_O_5_: 361.1763, found, 361.1761; ^1^H NMR (200 MHz, δ, CDCl_3_, TMS, PhNHNH_2_) 1.26 (s, 6H, (C*H*_3_)(CH_3_)CN(OH)C(CH_3_)(C*H*_3_)), 1.28 (s, 6H, (CH_3_)(C*H*_3_)CN(OH)C(C*H*_3_)(CH_3_)), 1.74 (t, *J* = 12.0 Hz, 2H, *H*HC-CH(O-)-CH*H*), 2.03 (ddd, *J* = 11.2 Hz, *J* = 4.4 Hz, *J* = 1.4 Hz, 2H, H*H*C-CH(O-)-C*H*H), 2.51 (ArCH_3_), 5.22 (tt, *J* = 11.3 Hz, *J* = 4.3 Hz, 1H, CH-OC(=O)), 6.42 (d, *J* = 15.9 Hz, 1H, Ar-CH=C*H*-COO), 7.90 (d, *J* = 15.9 Hz, 1H, Ar-C*H*=CH-COO); ^13^C NMR (50 MHz, δ, CDCl_3_, TMS, PhNHNH_2_) 20.10 (ArCH_3_), 20.90 (2×CH_3_), 31.66 (2×CH_3_), 43.81 (2×CH_2_), 60.32 (2×*C*(CH_3_)_2_), 67.47 (CH-O-), 121.62 (CH), 123.52 (CH), 125.72 (CH), 127.52 (CH), 128.95 (C), 139.22 (C), 139.93 (C), 140.21 (CH), 165.86 (C=O); IR (cm^−1^, KBr) 1715, 1519, 1347, 1170.

### 2,2,6,6-Tetramethyl-1-oxyl-4-piperidyl 3-*E*-(2-chloro-4-nitrophenyl)acrylate (**5i**)

56.2%; mp 107–110 °C; MS (EI, 70 eV, *m*/*z*, int [%]) 383 (1), 382 (1), 381 (2, M^+^), 212 (14), 211 (9), 210 (41, ArCH=CHCO), 194 (4), 192 (8), 166 (4), 164 (11), 154 (15), 140 (17), 139 (14), 138 (9), 136 (23), 124 (89), 109 (100), 101 (13), 100 (19), 99 (7), 98 (9), 95 (8), 89 (13), 82 (19), 81 (22), 75 (12), 74 (12), 69 (19), 68 (13), 67 (23), 57 (14), 56 (17), 55 (28), 43 (18), 41 (58); MS (ESI, *m*/*z*, int [%]): 404 (15, M + Na), 249 (100), 242 (15); HRMS (ESI): calcd for C_18_H_22_N_2_O_5_ClNa: 404.1115, found, 404.1131; ^1^H NMR (200 MHz, δ, CDCl_3_, TMS, PhNHNH_2_) 1.25, 1.27, 1.29 (3s, 12H, 4×CH_3_), 1.75 (m, 2H, *H*HC-CH(O-)-CH*H*), 2.01 (m, 2H, H*H*C-CH(O-)-C*H*H), 5.22 (tt, *J* = 11.3 Hz, *J* = 4.3 Hz, 1H, C*H*-OC(=O)), 6.51 (d, *J* = 16.0 Hz, 1H, Ar-CH=C*H*-COO), 8.03 (d, *J* = 16.0 Hz, 1H, Ar-C*H*=CH-COO); ^13^C NMR (50 MHz, δ, CDCl_3_, TMS, PhNHNH_2_) 20.88 (2×CH_3_), 31.67 (2×CH_3_), 43.75 (2×CH_2_), 60.33 (2×*C*(CH_3_)_2_), 67.72 (CH-O-), 122.22 (CH), 125.02 (CH), 125.58 (CH), 128.53 (C), 128.87 (C), 128.90 (CH), 138.45 (CH), 139.10 (C), 165.31 (C=O); IR (cm^−1^, KBr) 2976, 1712, 1523, 1346, 1330, 1194, 1180, 990.

## Supporting Information

File 1 EIMS, ESIMS, ^1^H NMR, ^13^C NMR, and IR spectra of **3** and **5a**–**5i**.
