# Supplementary material for: Reactions of nitroxides 15. Cinnamates bearing a nitroxyl moiety synthesized using a Mizoroki–Heck cross-coupling reaction
Source: Beilstein J Org Chem. 2015 Jul 13;11:1155–62. doi: 10.3762/bjoc.11.130 (PMC4505299; doi:10.3762/bjoc.11.130)
Supplement: File 1 — EIMS, ESIMS, 1H NMR, 13C NMR, and IR spectra of 3 and 5a–5i. [file Beilstein_J_Org_Chem-11-1155-s001.pdf]

Supporting Information

for

Reactions of nitroxides 15. Cinnamates bearing a nitroxyl moiety synthesized using a Mizoroki–Heck cross-coupling reaction

Jerzy Zakrzewski*, Bogumiła Huras

Address: Institute of Industrial Organic Chemistry, Annopol 6, 03-236 Warsaw, Poland

Email: Jerzy Zakrzewski* - zakrzewski@ipo.waw.pl

EIMS, ESIMS, ^{1}H NMR, ^{13}C NMR, and IR spectra of 3 and 5a–5i.

EIMS, ESIMS, ^{1}H NMR, ^{13}C NMR, and IR spectra of 3 and 5a–5i.

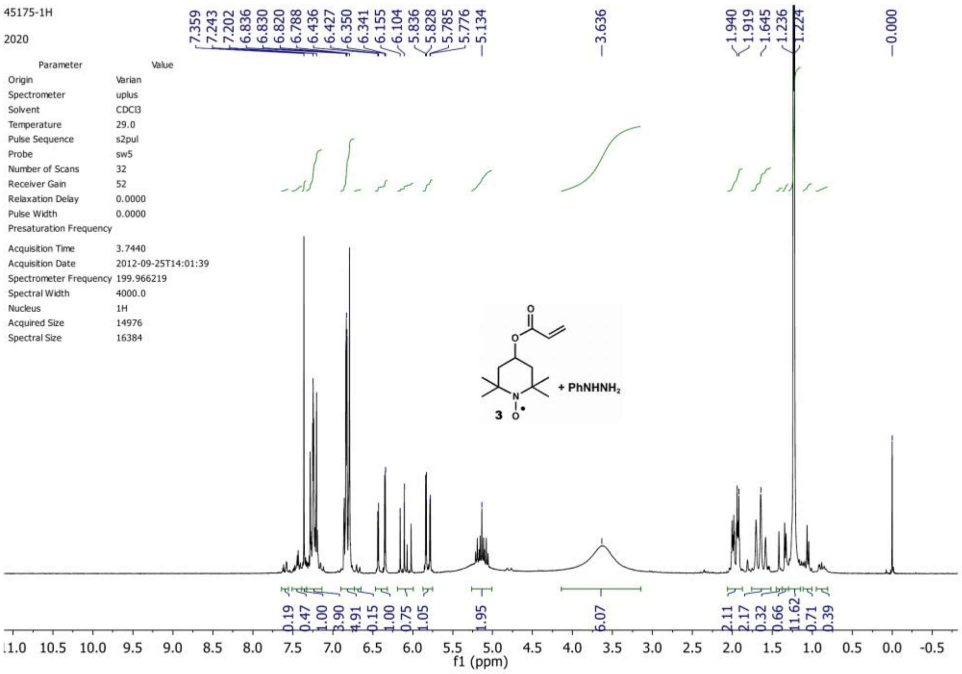

Fig. S1. 4-Acryloyloxy-2,2,6,6-tetramethylpiperidine-1-oxyl (**3**) + $C_6H_5HNH_2$, ^{1}H NMR

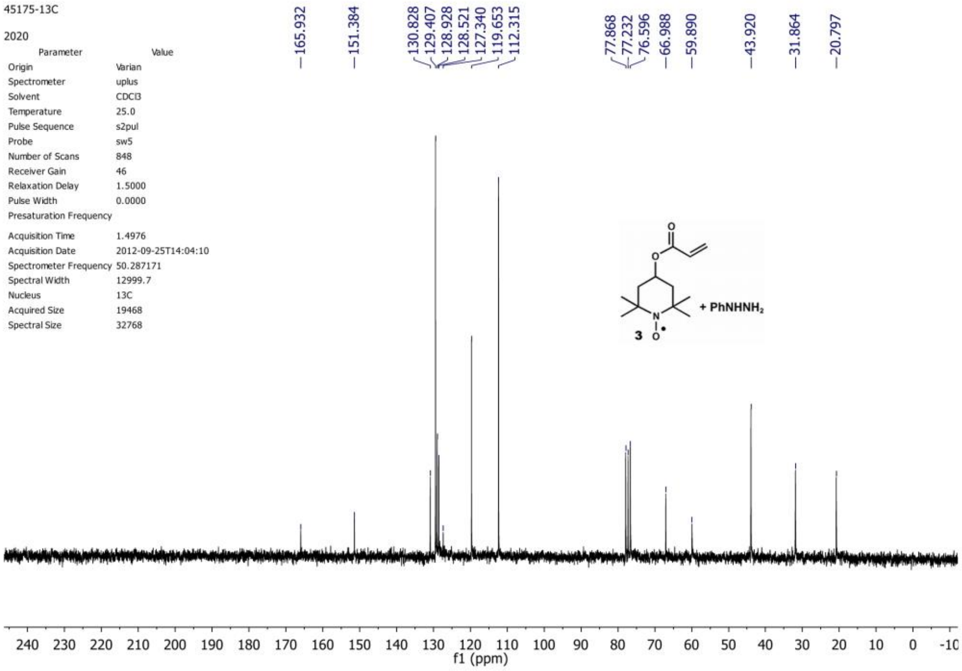

Fig. S2. 4-Acryloyloxy-2,2,6,6-tetramethylpiperidine-1-oxyl (**3**) + $C_6H_5HNH_2$, ^{13}C NMR

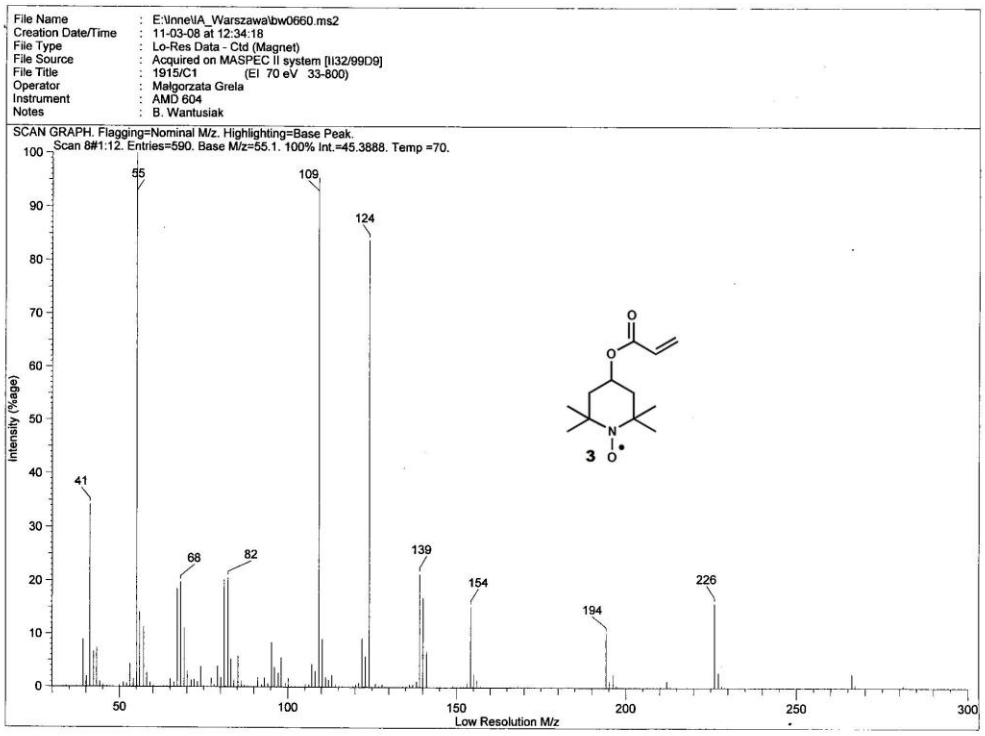

Fig. S3. 4-Acryloyloxy-2,2,6,6-tetramethylpiperidine-1-oxyl (**3**), EI MS

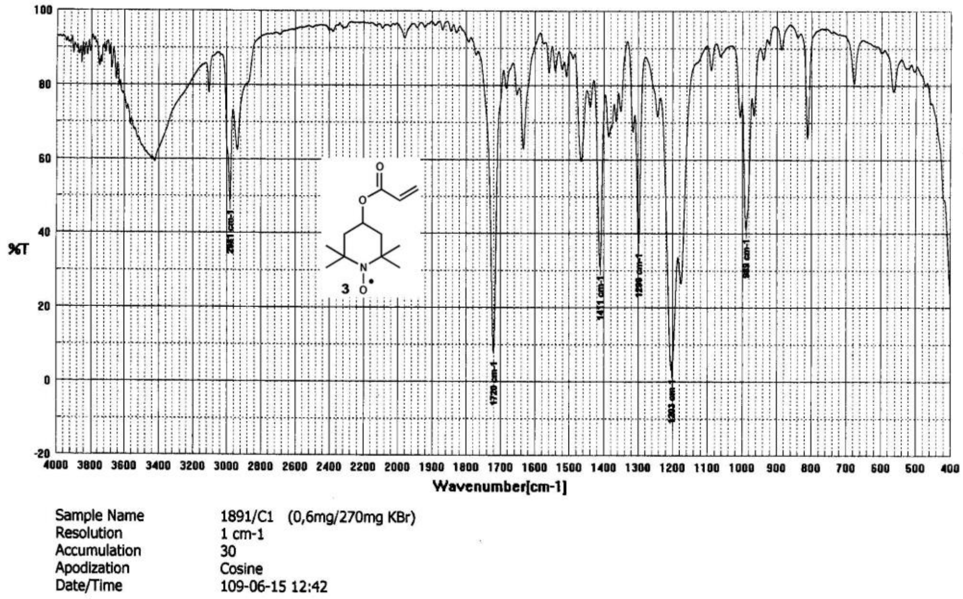

Fig. S4. 4-Acryloyloxy-2,2,6,6-tetramethylpiperidine-1-oxyl (**3**), IR

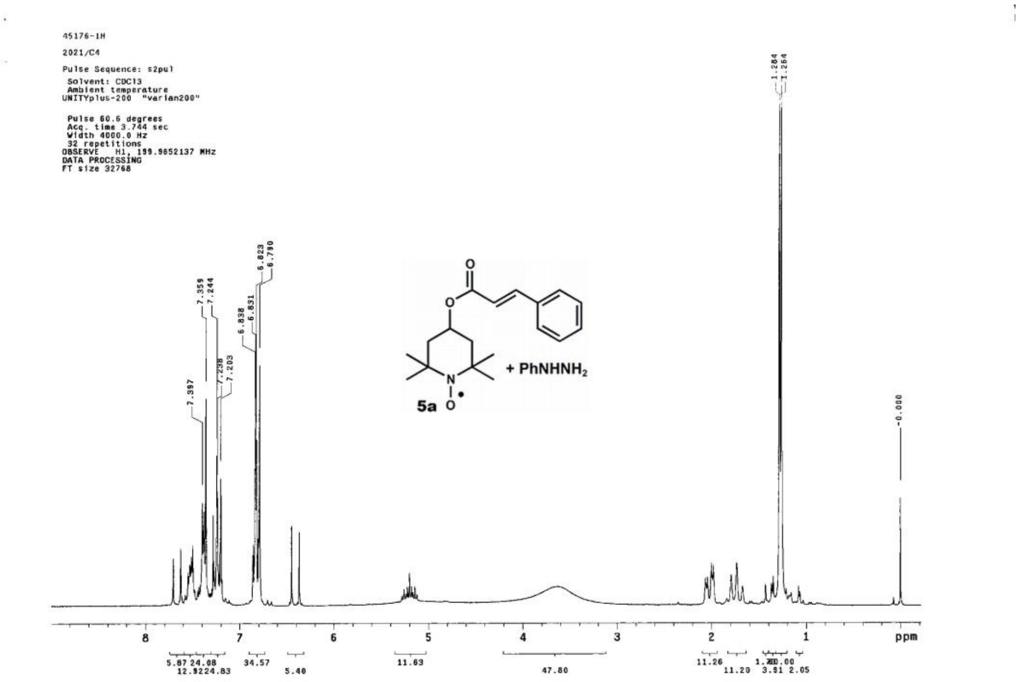

Fig. S5. 2,2,6,6-Tetramethyl-1-oxyl-4-piperidyl 3-*E*-phenylacrylate (**5a**) + $C_6H_5HNH_2$, ^{1}H NMR

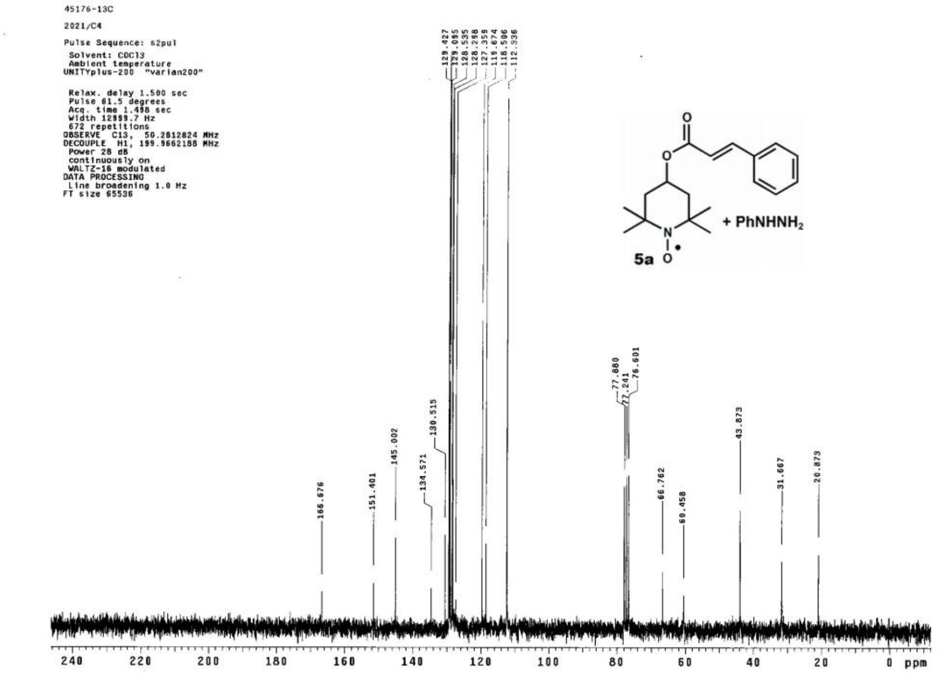

Fig. S6. 2,2,6,6-Tetramethyl-1-oxyl-4-piperidyl 3-*E*-phenylacrylate (**5a**) + $C_6H_5HNH_2$, ^{13}C NMR

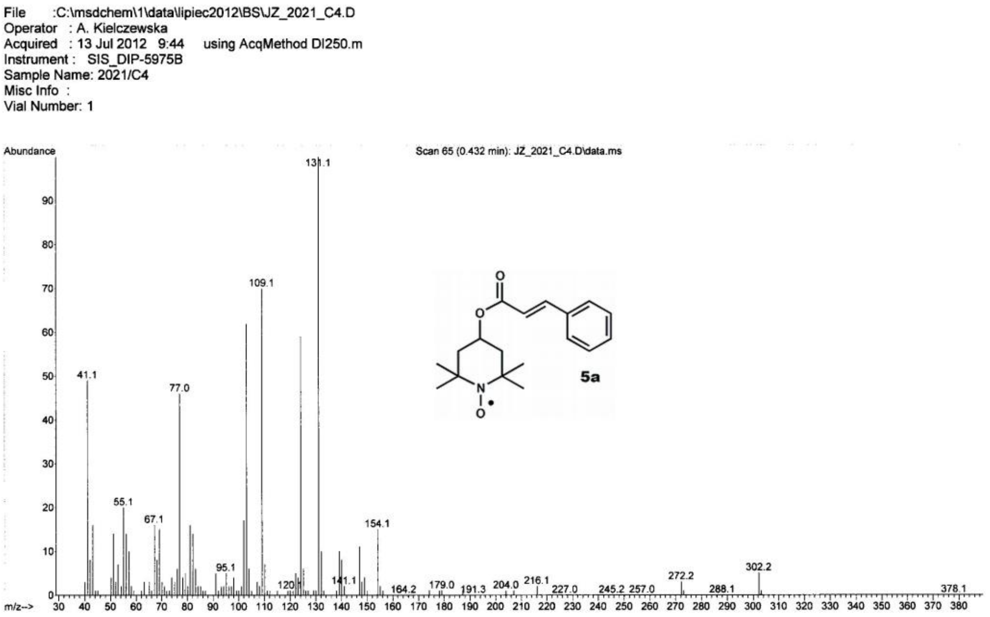

Fig. S7. 2,2,6,6-Tetramethyl-1-oxyl-4-piperidyl 3-*E*-phenylacrylate (**5a**), EI MS

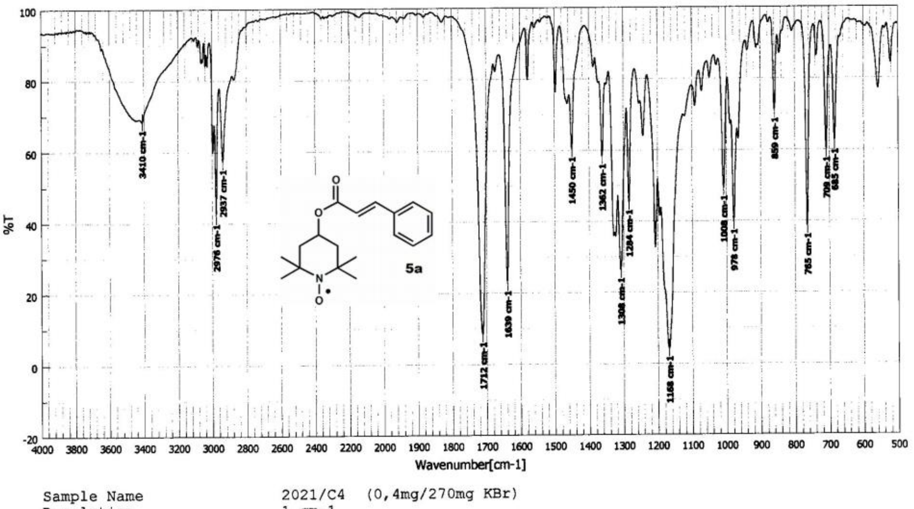


```
Sample Name      2021/C4  (0,4mg/270mg KBr)
Resolution       1 cm-1
Accumulation     30
Apodization      Cosine
Date/Time        112-07-13 10:15
```

Fig. S8. 2,2,6,6-Tetramethyl-1-oxyl-4-piperidyl 3-*E*-phenylacrylate (**5a**), IR

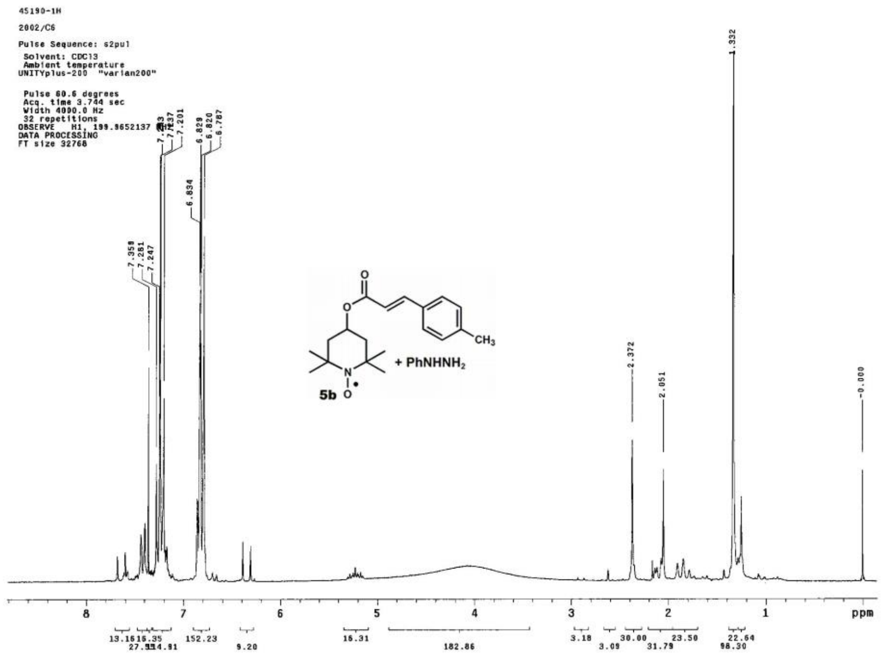

Fig. S9. 2,2,6,6-Tetramethyl-1-oxyl-4-piperidyl 3-*E*-(4-methylphenyl)acrylate (**5b**) + $C_6H_5NHNH_2$, ^{1}H NMR

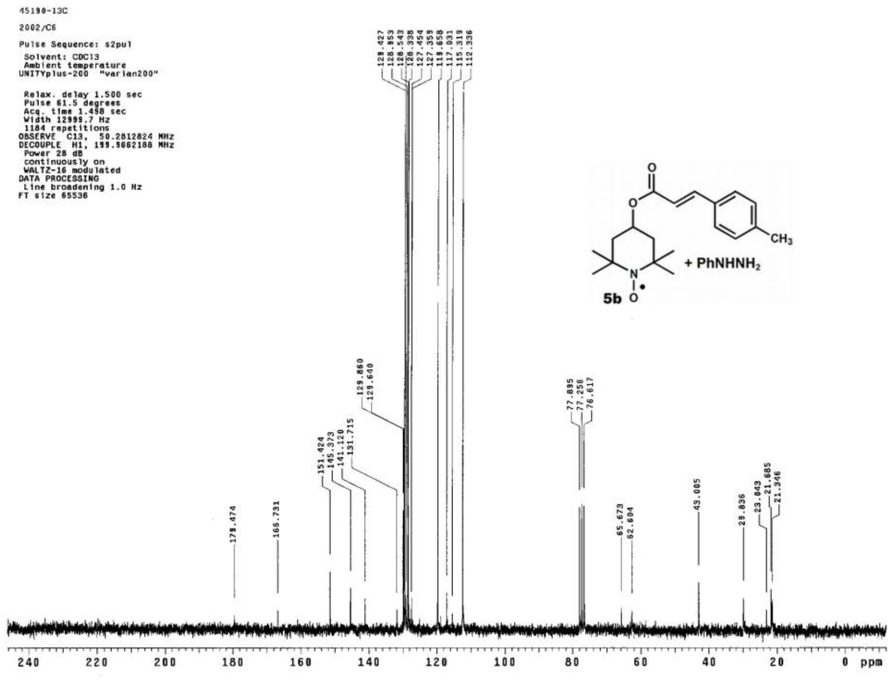

Fig. S10. 2,2,6,6-Tetramethyl-1-oxyl-4-piperidyl 3-*E*-(4-methylphenyl)acrylate (**5b**) + $C_6H_5NHNH_2$, ^{13}C NMR

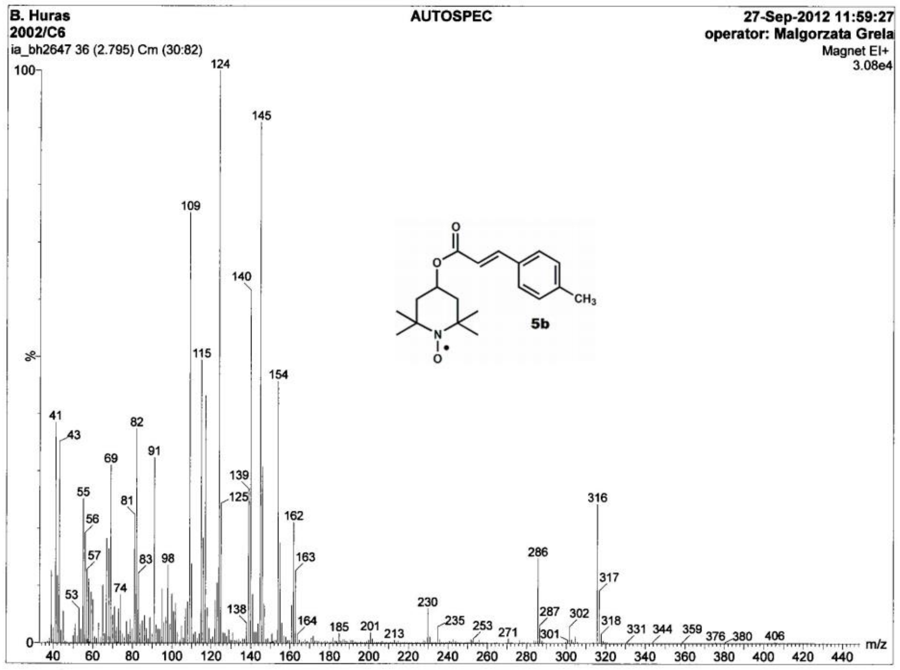

Fig. S11. 2,2,6,6-Tetramethyl-1-oxyl-4-piperidyl 3-*E*-(4-methylphenyl)acrylate (**5b**), EI MS

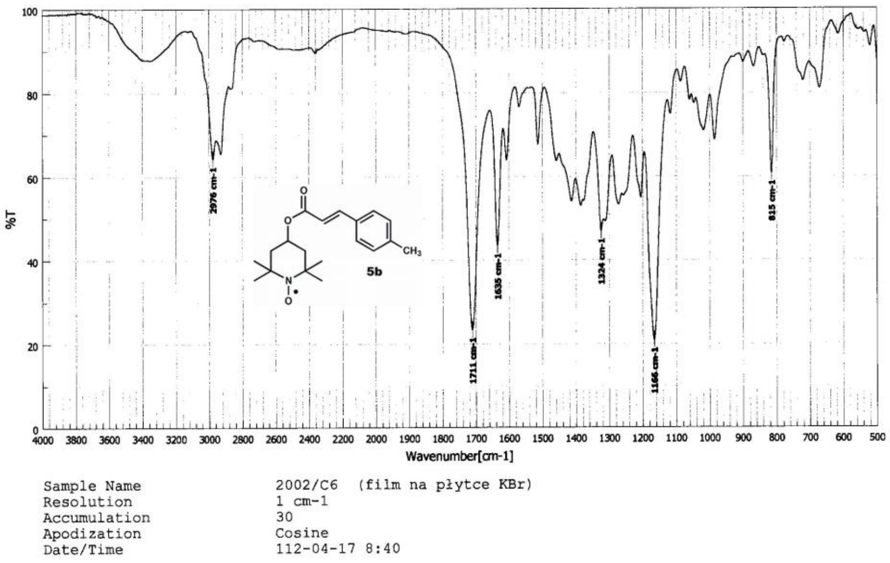

Fig. S12. 2,2,6,6-Tetramethyl-1-oxyl-4-piperidyl 3-*E*-(4-methylphenyl)acrylate (**5b**), IR

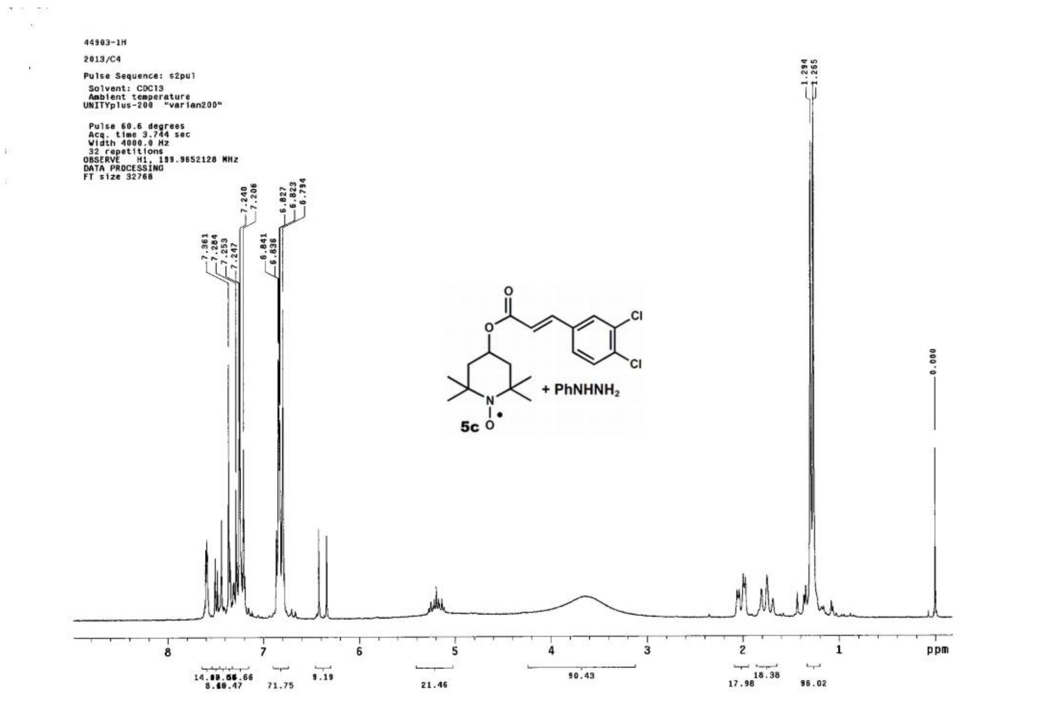

Fig. S13. 2,2,6,6-Tetramethyl-1-oxyl-4-piperidyl 3-*E*-(3,4-dichlorophenyl)acrylate (**5c**) + $C_6H_5NHNH_2$, ^{1}H NMR

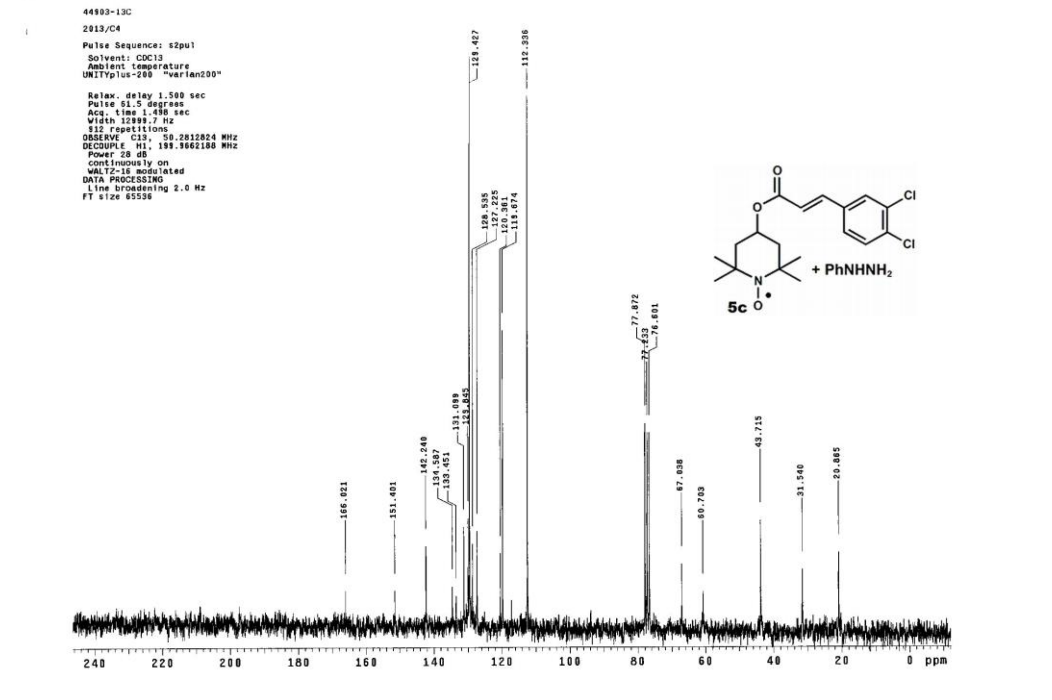

Fig. S14. 2,2,6,6-Tetramethyl-1-oxyl-4-piperidyl 3-*E*-(3,4-dichlorophenyl)acrylate (**5c**) + $C_6H_5NHNH_2$, ^{13}C NMR

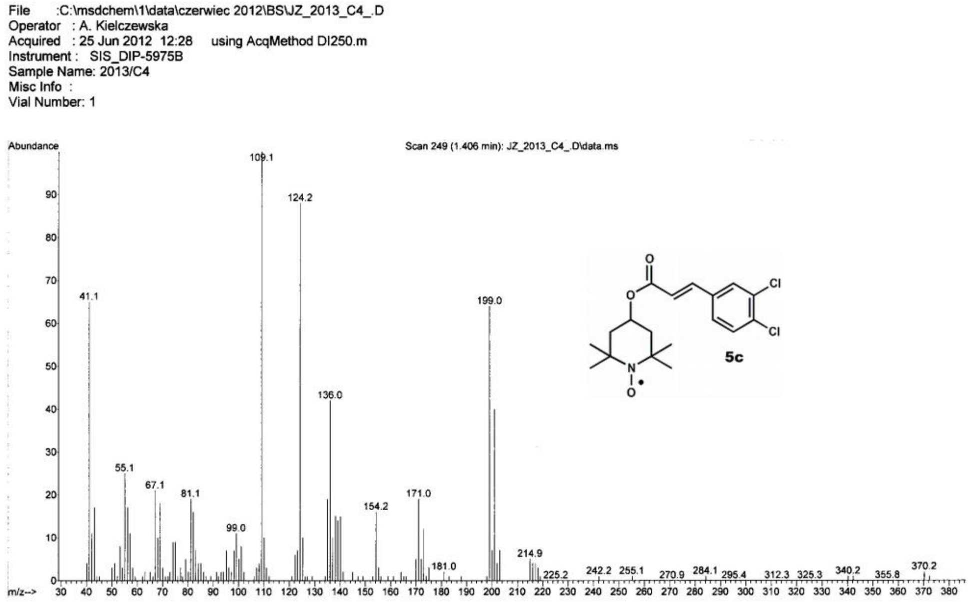

Fig. S15. 2,2,6,6-Tetramethyl-1-oxyl-4-piperidyl 3-*E*-(3,4-dichlorophenyl)acrylate (**5c**), EI MS

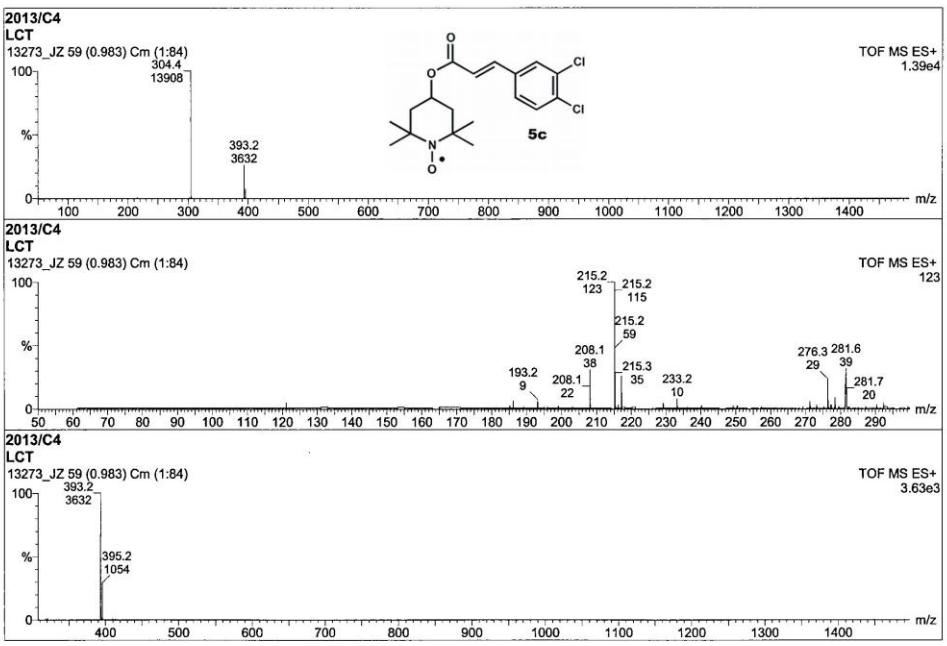

Fig. S16. 2,2,6,6-Tetramethyl-1-oxyl-4-piperidyl 3-*E*-(3,4-dichlorophenyl)acrylate (**5c**), ESI MS

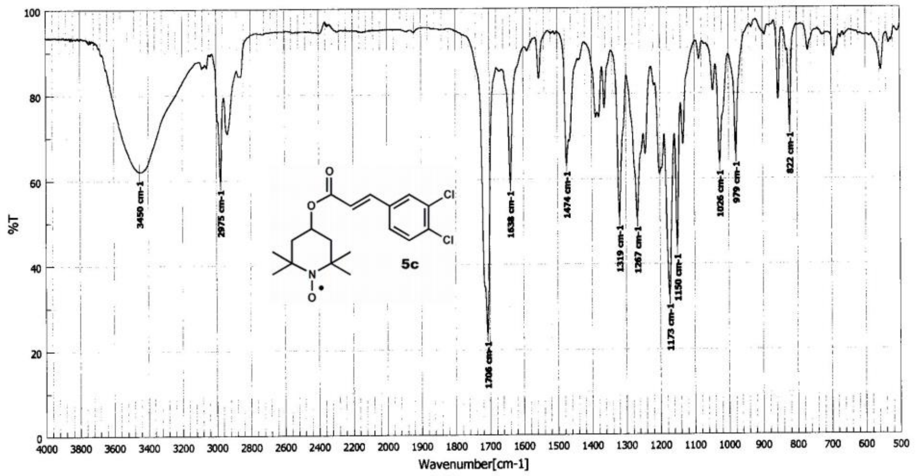

Sample Name 2013/C4 (0,5mg/270mg KBr)
Resolution 1 cm-1
Accumulation 30
Apodization Cosine
Date/Time 112-06-21 12:14

Fig. S17. 2,2,6,6-Tetramethyl-1-oxyl-4-piperidyl 3-*E*-(3,4-dichlorophenyl)acrylate (**5c**), IR

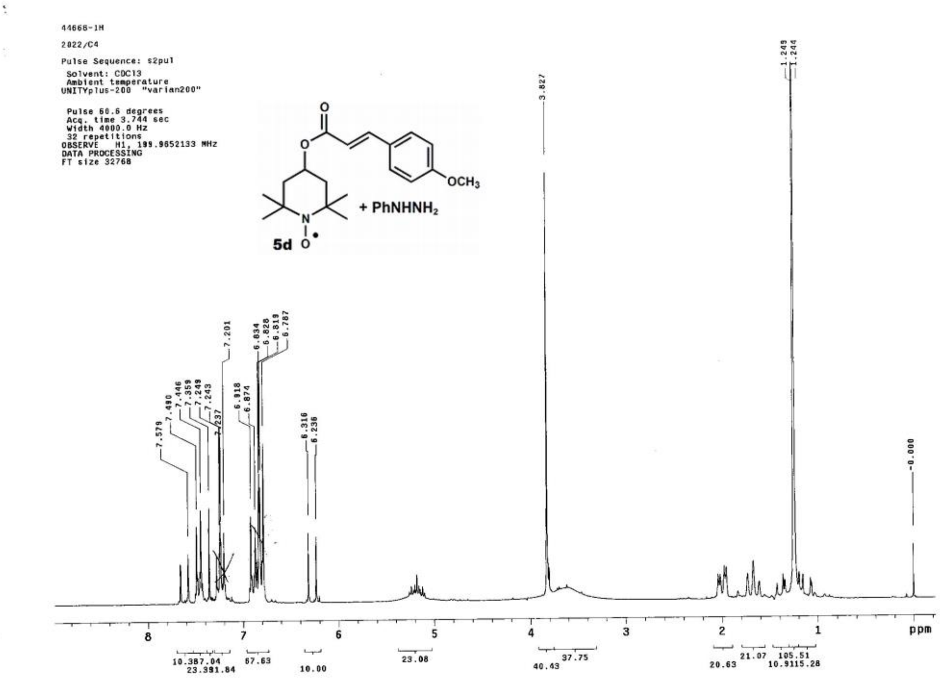

Fig. S18. 2,2,6,6-Tetramethyl-1-oxyl-4-piperidyl 3-*E*-(4-methoxyphenyl)acrylate (**5d**) + $C_6H_5NHNH_2$, ^1H NMR

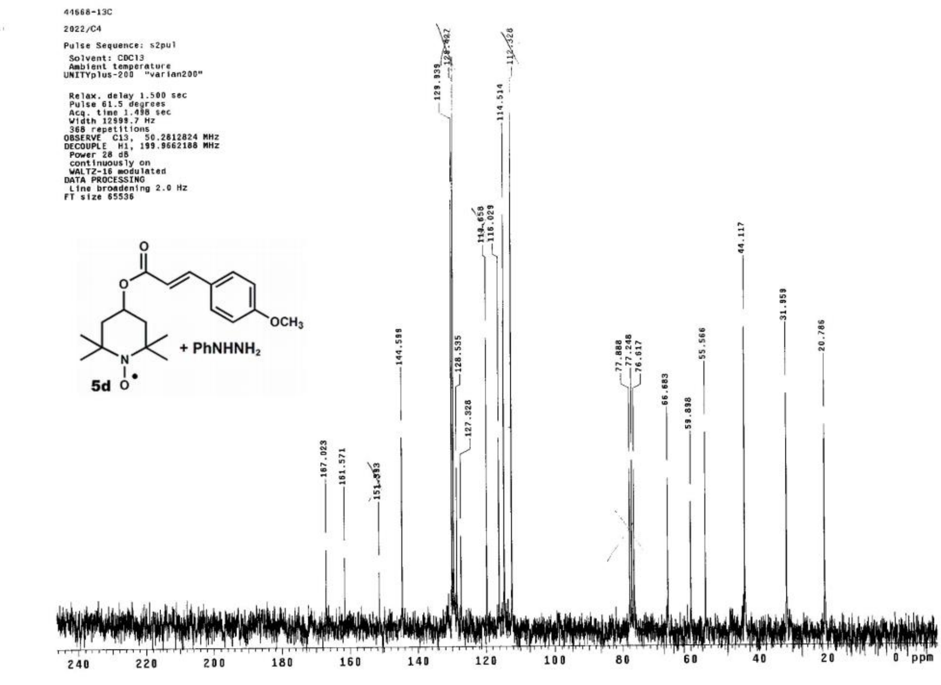

Fig. S19. 2,2,6,6-Tetramethyl-1-oxyl-4-piperidyl 3-*E*-(4-methoxyphenyl)acrylate (**5d**) + $C_6H_5NHNH_2$, ^{13}C NMR

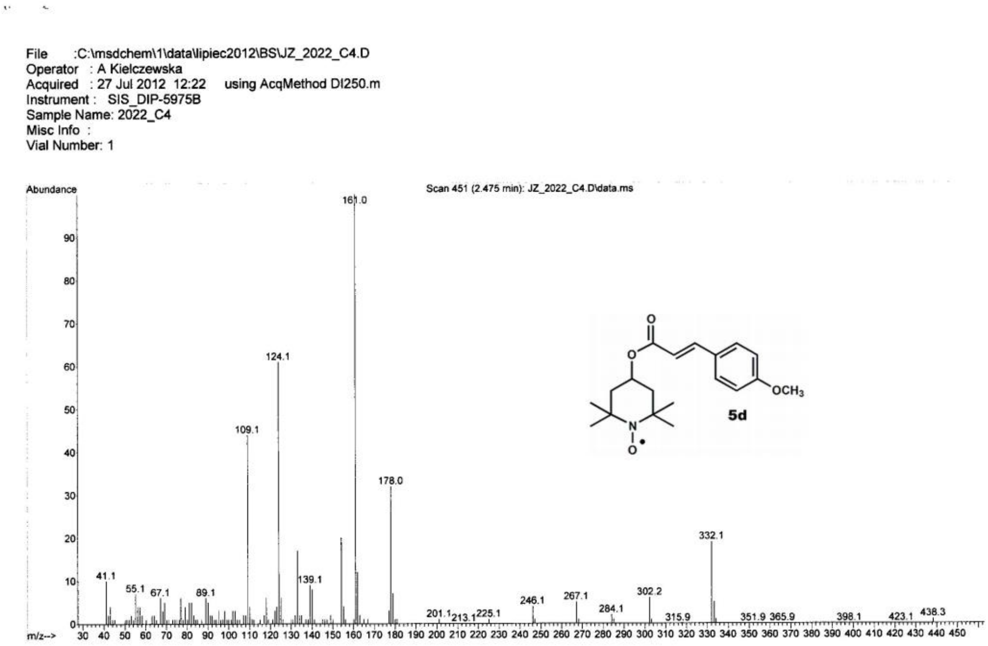

Fig. S20. 2,2,6,6-Tetramethyl-1-oxyl-4-piperidyl 3-*E*-(4-methoxyphenyl)acrylate (**5d**), EI MS

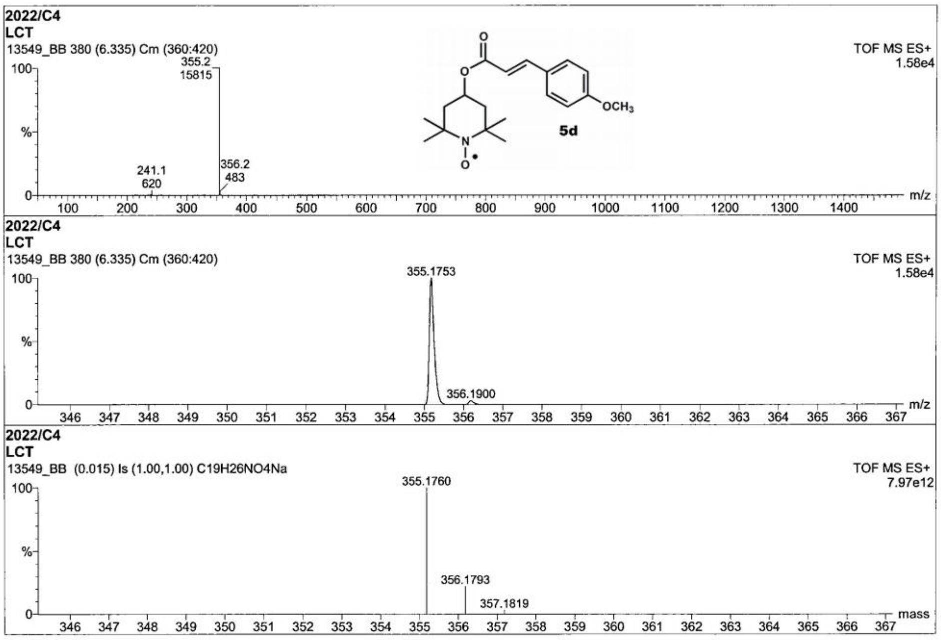

Fig. S21. 2,2,6,6-Tetramethyl-1-oxyl-4-piperidyl 3-*E*-(4-methoxyphenyl)acrylate (**5d**), ESI MS

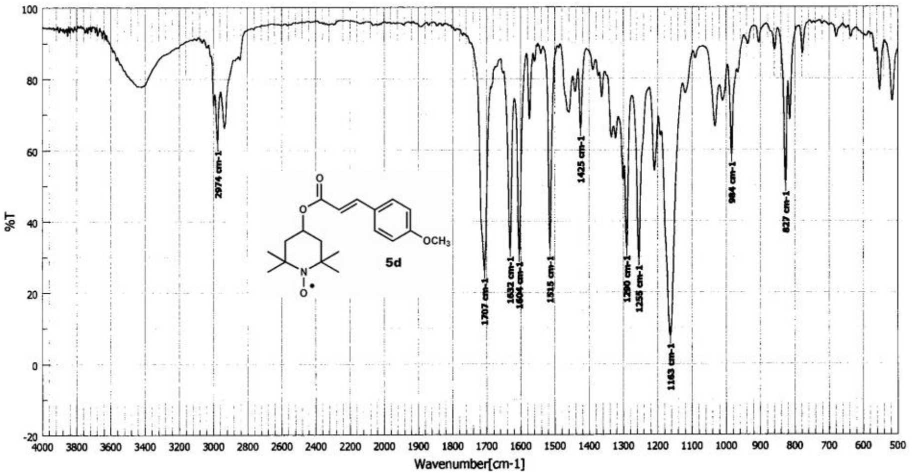

Sample Name	2022/C4 (0,5mg/270mg KBr)
Resolution	1 cm-1
Accumulation	30
Apodization	Cosine
Date/Time	112-07-31 10:07

Fig. S22. 2,2,6,6-Tetramethyl-1-oxyl-4-piperidyl 3-*E*-(4-methoxyphenyl)acrylate (**5d**), IR

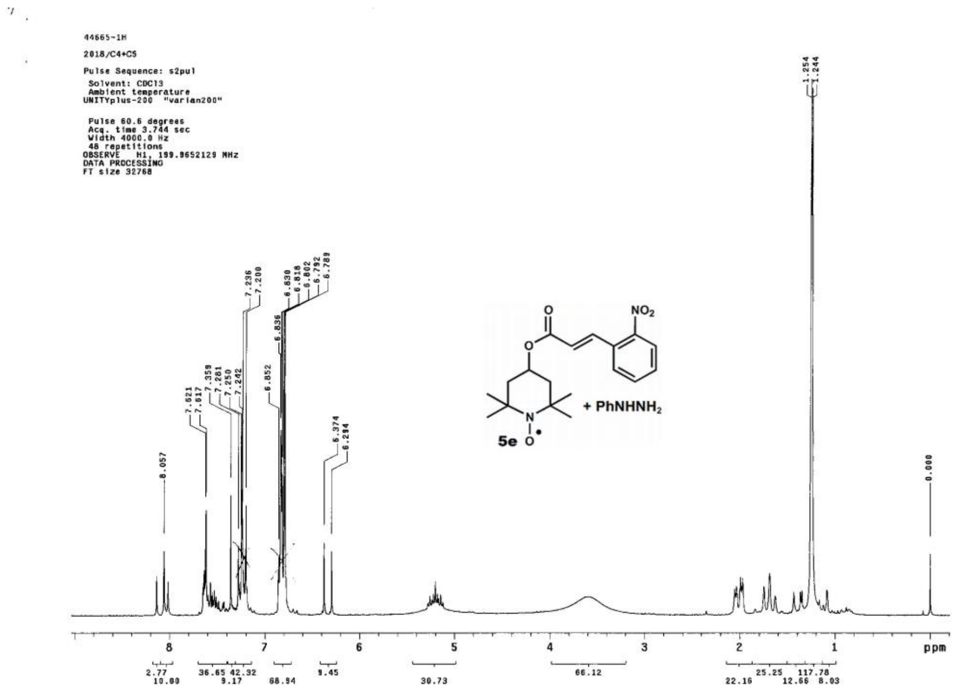

Fig. S23. 2,2,6,6-Tetramethyl-1-oxyl-4-piperidyl 3-*E*-(2-nitrophenyl)acrylate (**5e**) + $C_6H_5NHNH_2$, ^1H NMR

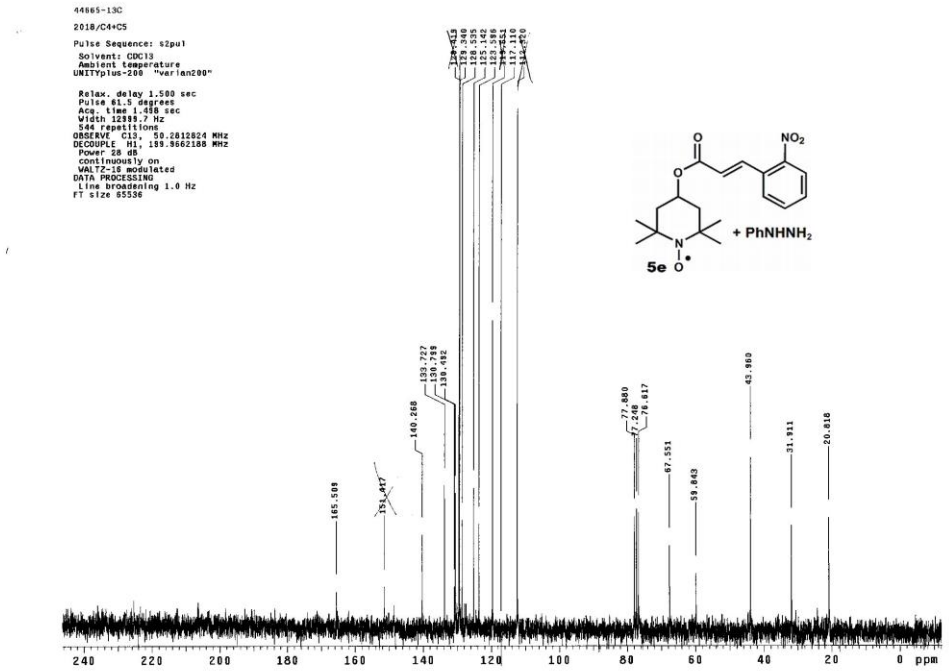

Fig. S24. 2,2,6,6-Tetramethyl-1-oxyl-4-piperidyl 3-*E*-(2-nitrophenyl)acrylate (**5e**) + $C_6H_5NHNH_2$, ^{13}C NMR

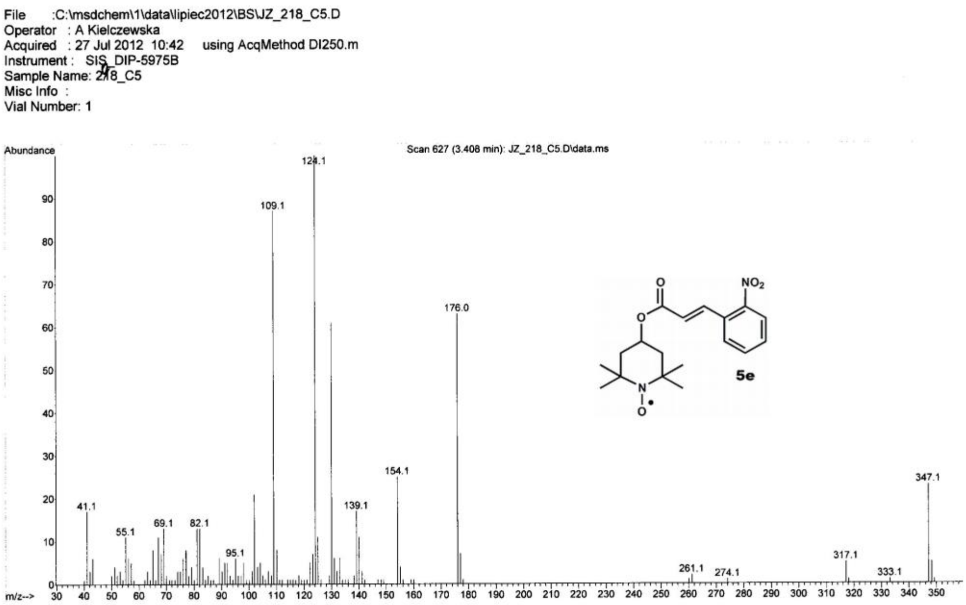

Fig. *S*25. 2,2,6,6-Tetramethyl-1-oxyl-4-piperidyl 3-*E*-(2-nitrophenyl)acrylate (**5e**), EI MS

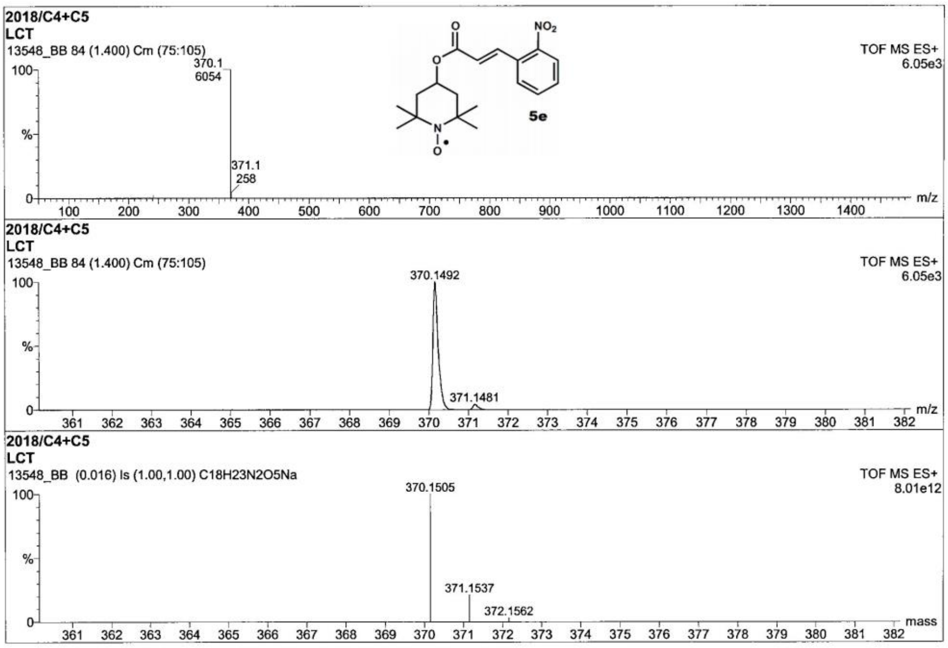

Fig. *S*26. 2,2,6,6-Tetramethyl-1-oxyl-4-piperidyl 3-*E*-(2-nitrophenyl)acrylate (**5e**), ESI MS

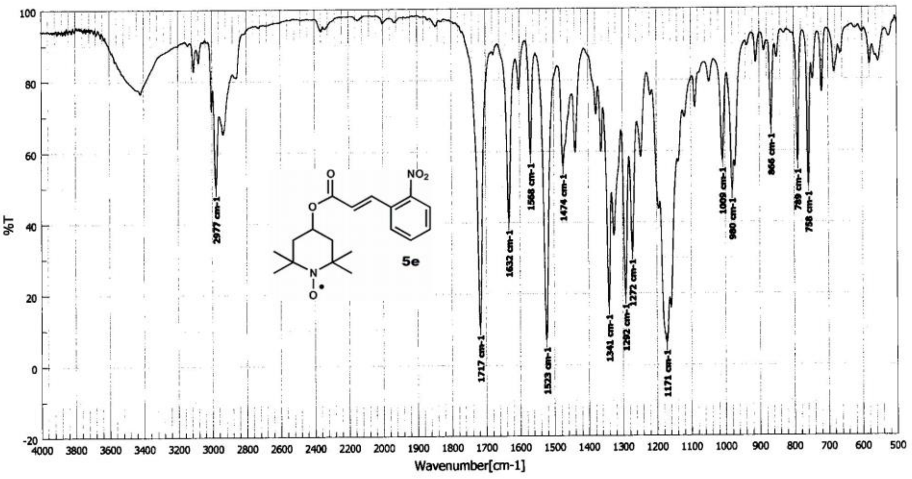


```
Sample Name        2018/C5  (0,8mg/270mg KBr)
Resolution         1 cm-1
Accumulation       30
Apodization        Cosine
Date/Time          112-07-31 14:04
```

Fig. S27. 2,2,6,6-Tetramethyl-1-oxyl-4-piperidyl 3-*E*-(2-nitrophenyl)acrylate (**5e**), IR

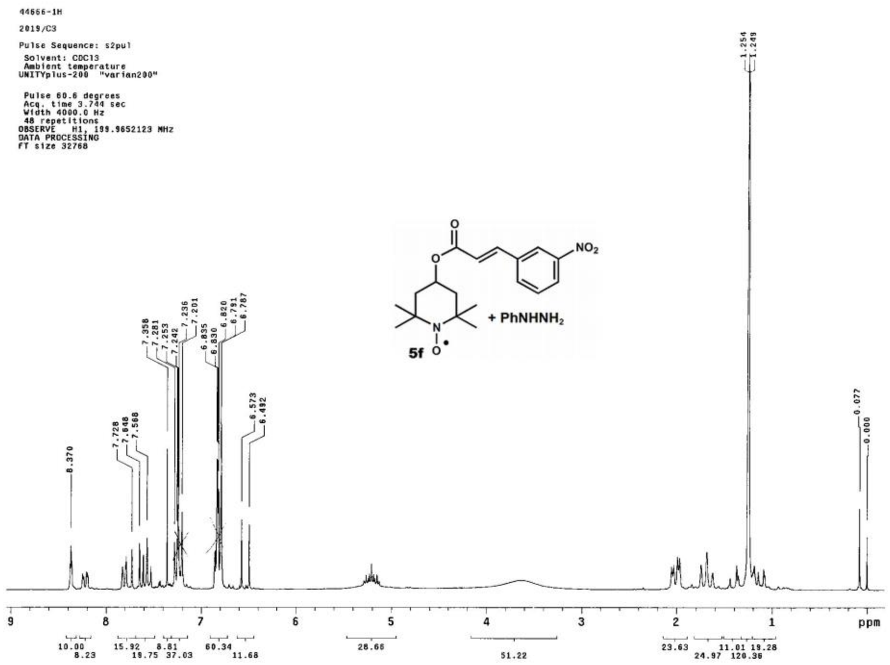

Fig. *S*28. 2,2,6,6-Tetramethyl-1-oxyl-4-piperidyl 3-*E*-(3-nitrophenyl)acrylate (**5f**) + $C_6H_5NHNH_2$, ^{1}H NMR

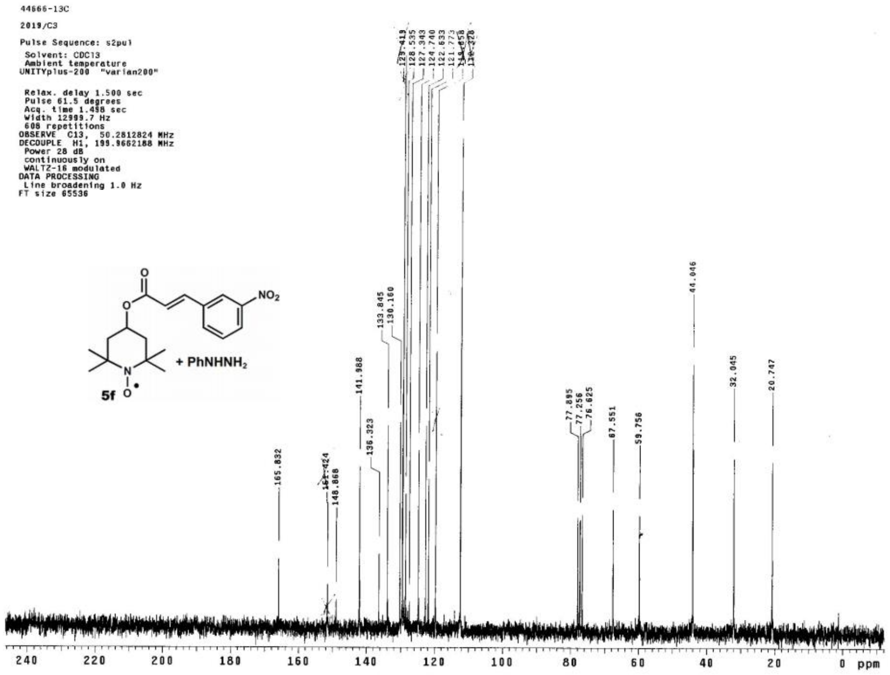

Fig. *S*29. 2,2,6,6-Tetramethyl-1-oxyl-4-piperidyl 3-*E*-(3-nitrophenyl)acrylate (**5f**) + $C_6H_5NHNH_2$, ^{13}C NMR

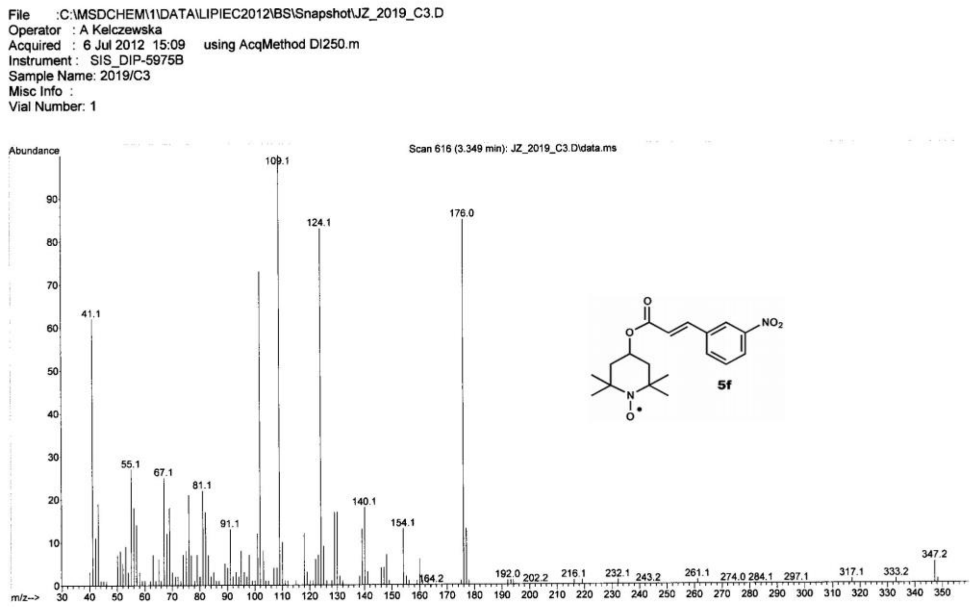

Fig. S30. 2,2,6,6-Tetramethyl-1-oxyl-4-piperidyl 3-*E*-(3-nitrophenyl)acrylate (**5f**), EI MS

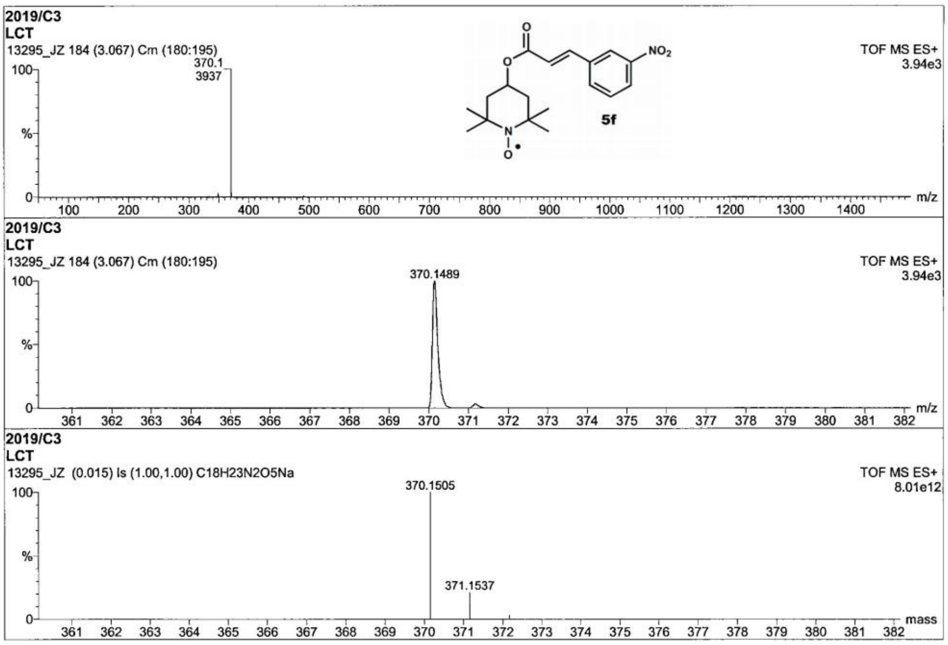

Fig. S31. 2,2,6,6-Tetramethyl-1-oxyl-4-piperidyl 3-*E*-(3-nitrophenyl)acrylate (**5f**), ESI MS

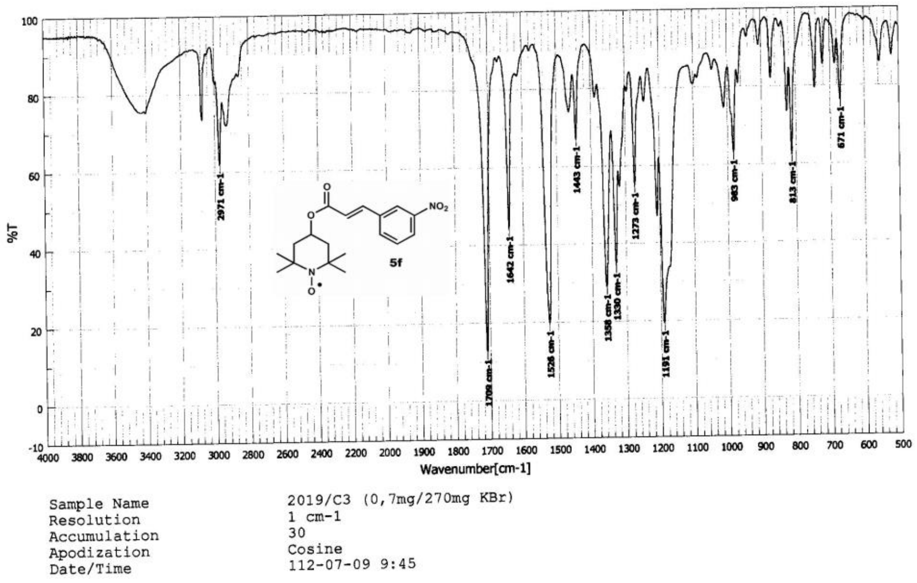

Fig. S32. 2,2,6,6-Tetramethyl-1-oxyl-4-piperidyl 3-*E*-(3-nitrophenyl)acrylate (**5f**), IR

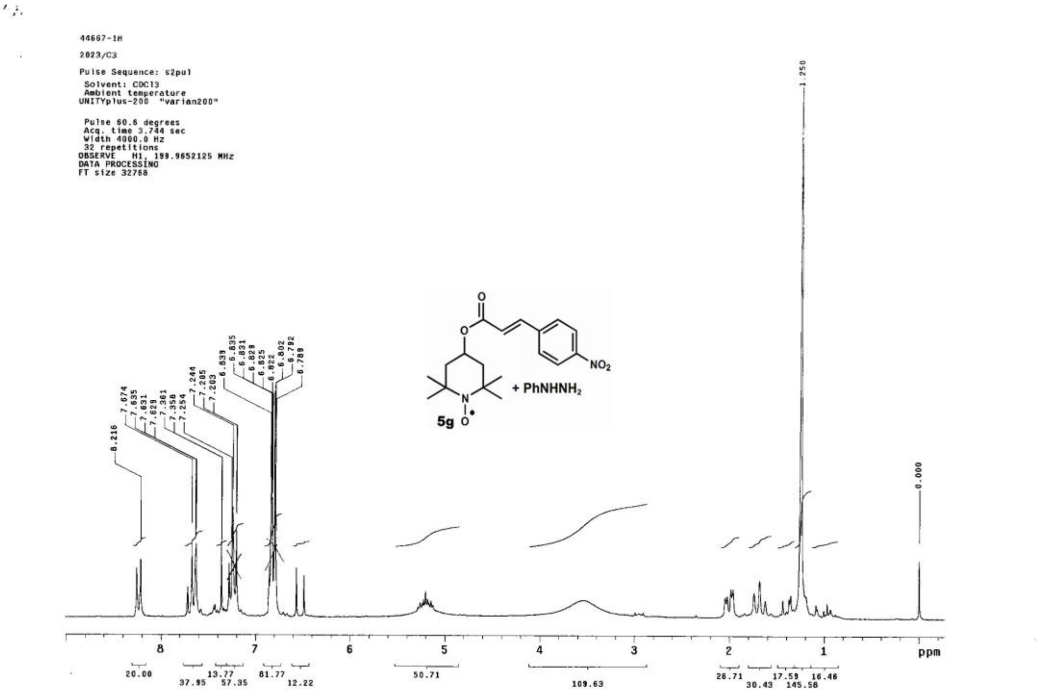

Fig. S33. 2,2,6,6-Tetramethyl-1-oxyl-4-piperidyl 3-*E*-(4-nitrophenyl)acrylate (**5g**) + $C_6H_5NHNH_2$, ^{1}H NMR

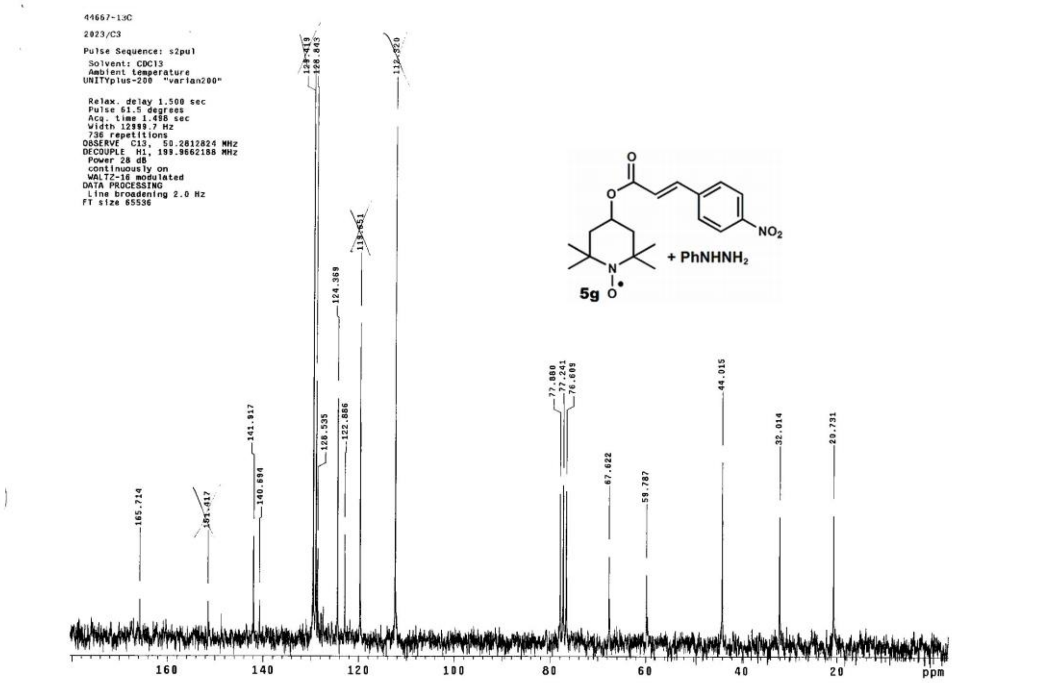

Fig. S34. 2,2,6,6-Tetramethyl-1-oxyl-4-piperidyl 3-*E*-(4-nitrophenyl)acrylate (**5g**) + $C_6H_5NHNH_2$, ^{13}C NMR

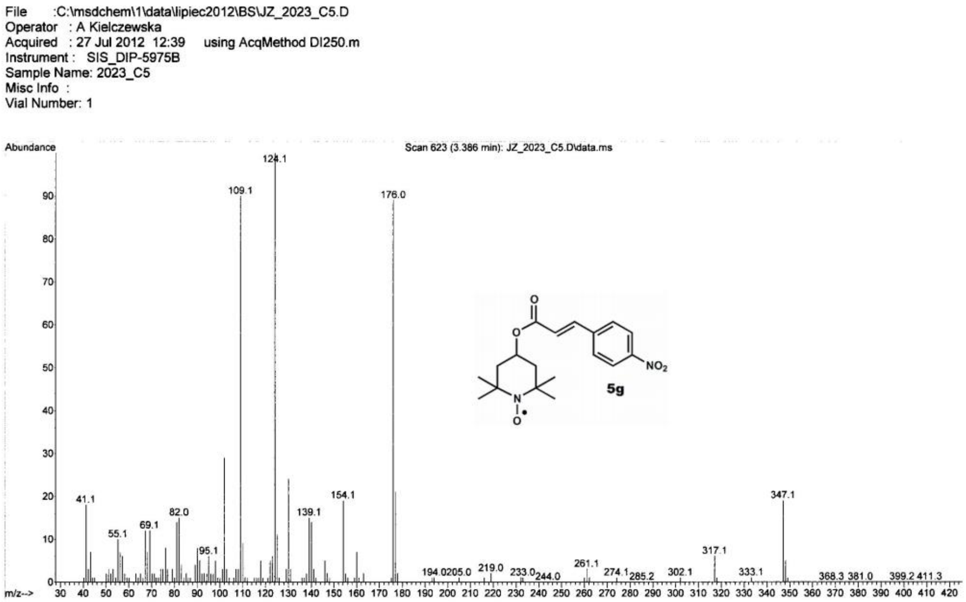

Fig. S35. 2,2,6,6-Tetramethyl-1-oxyl-4-piperidyl 3-*E*-(4-nitrophenyl)acrylate (**5g**), EI MS

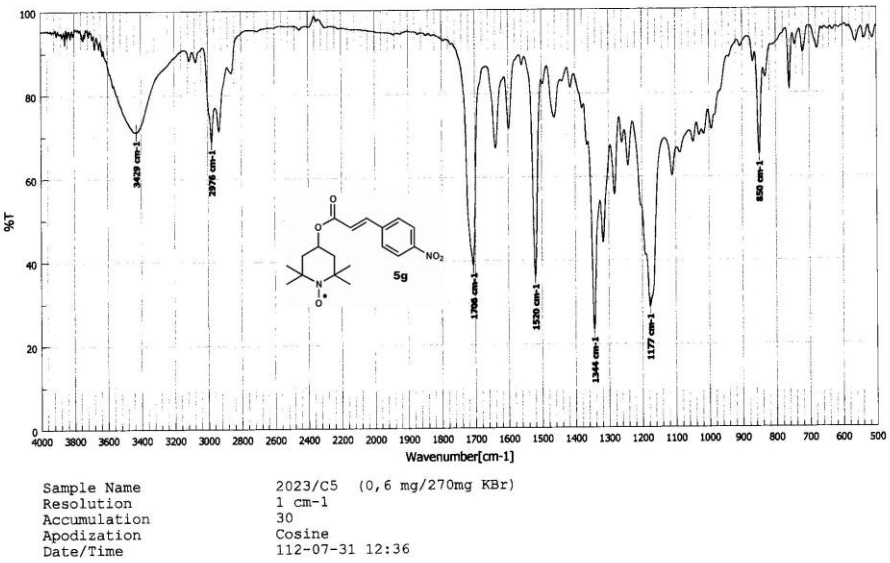

Fig. S36. 2,2,6,6-Tetramethyl-1-oxyl-4-piperidyl 3-*E*-(4-nitrophenyl)acrylate (**5g**), IR

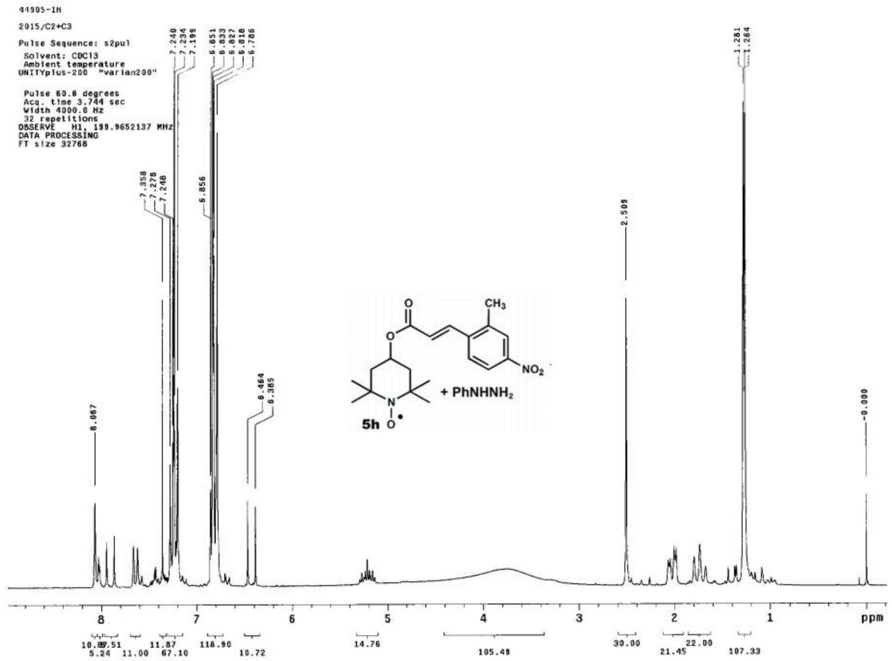

Fig. S37. 2,2,6,6-Tetramethyl-1-oxyl-4-piperidyl 3-*E*-(2-methyl-4-nitrophenyl)acrylate (**5h**) + $C_6H_5NHNH_2$, ^{1}H NMR

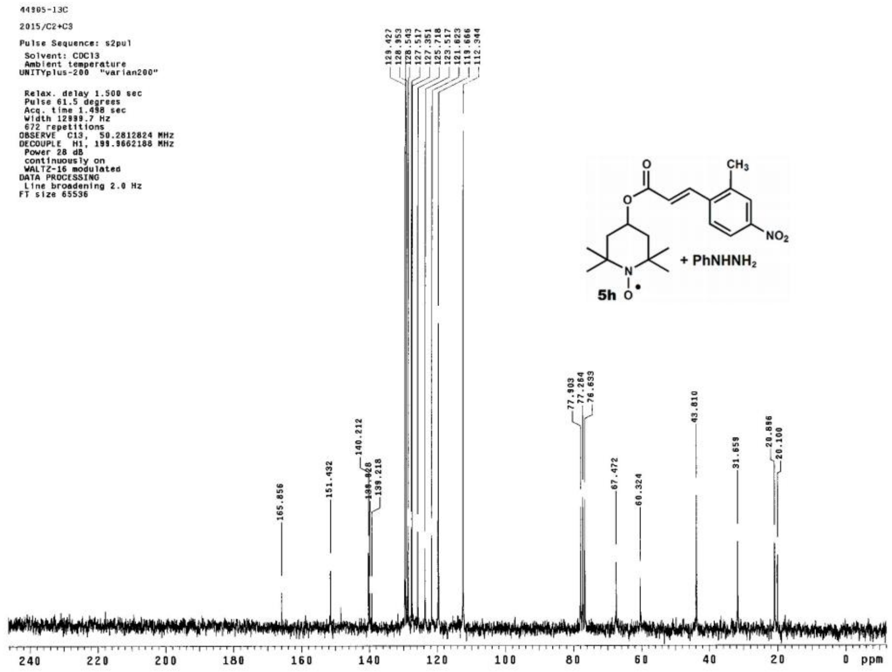

Fig. S38. 2,2,6,6-Tetramethyl-1-oxyl-4-piperidyl 3-*E*-(2-methyl-4-nitrophenyl)acrylate (**5h**) + $C_6H_5NHNH_2$, ^{13}C NMR

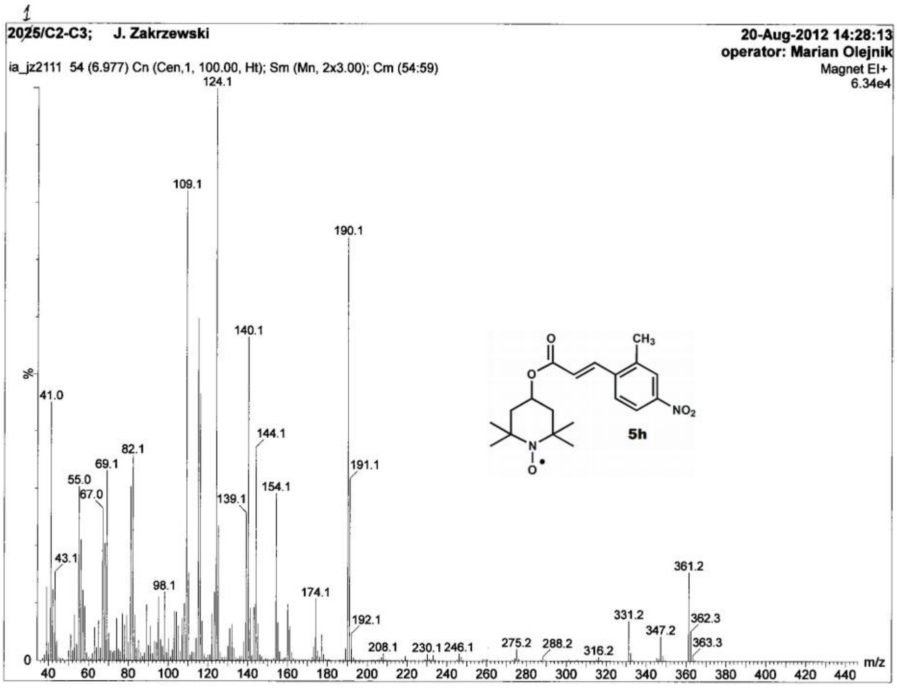

Fig. S39. 2,2,6,6-Tetramethyl-1-oxyl-4-piperidyl 3-*E*-(2-methyl-4-nitrophenyl)acrylate (**5h**), EI MS

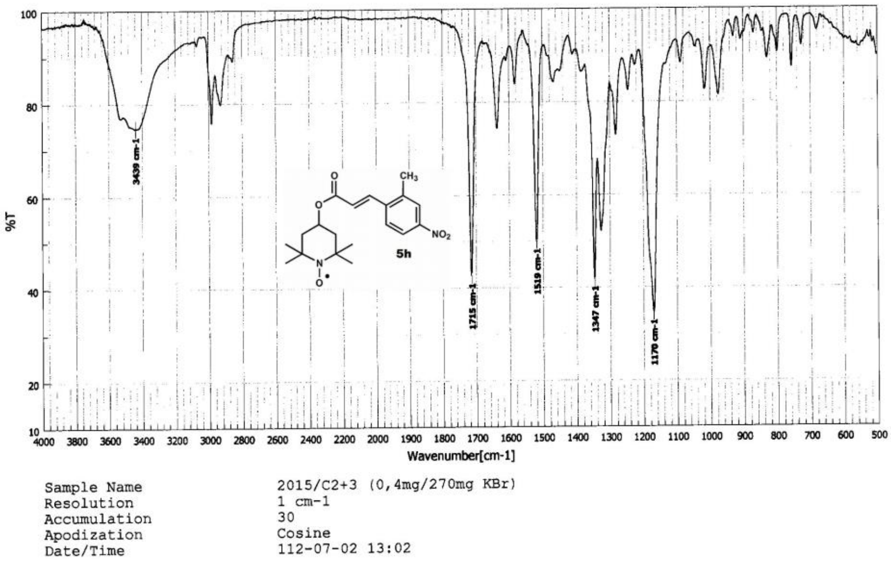

Fig. S40. 2,2,6,6-Tetramethyl-1-oxyl-4-piperidyl 3-*E*-(2-methyl-4-nitrophenyl)acrylate (**5h**), IR

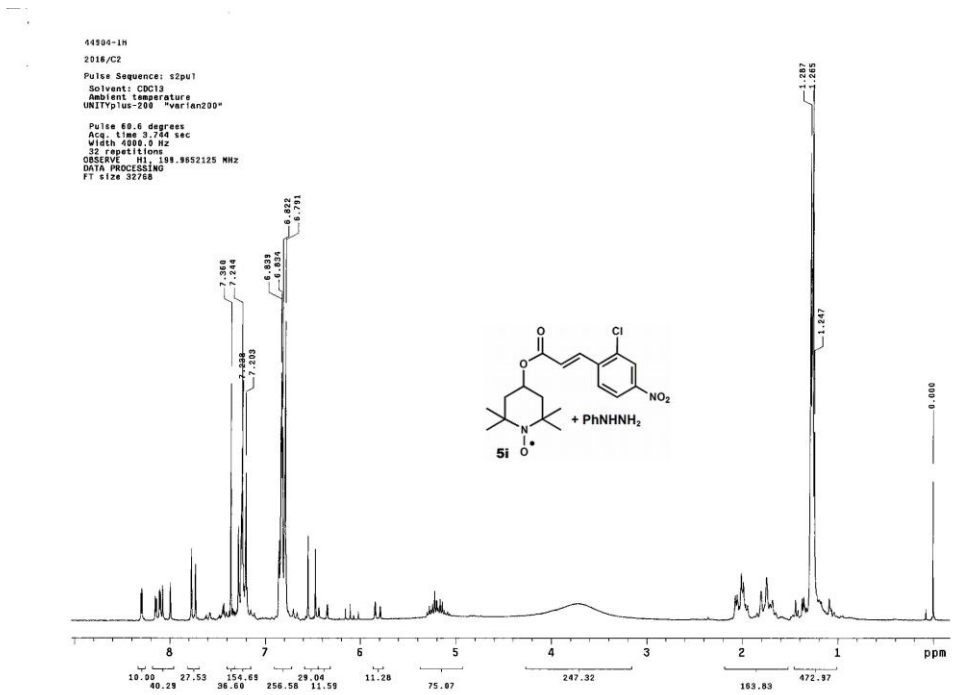

Fig. S41. 2,2,6,6-Tetramethyl-1-oxyl-4-piperidyl 3-*E*-(2-chloro-4-nitrophenyl)acrylate (**5i**) + $C_6H_5NHNH_2$, ^{1}H NMR

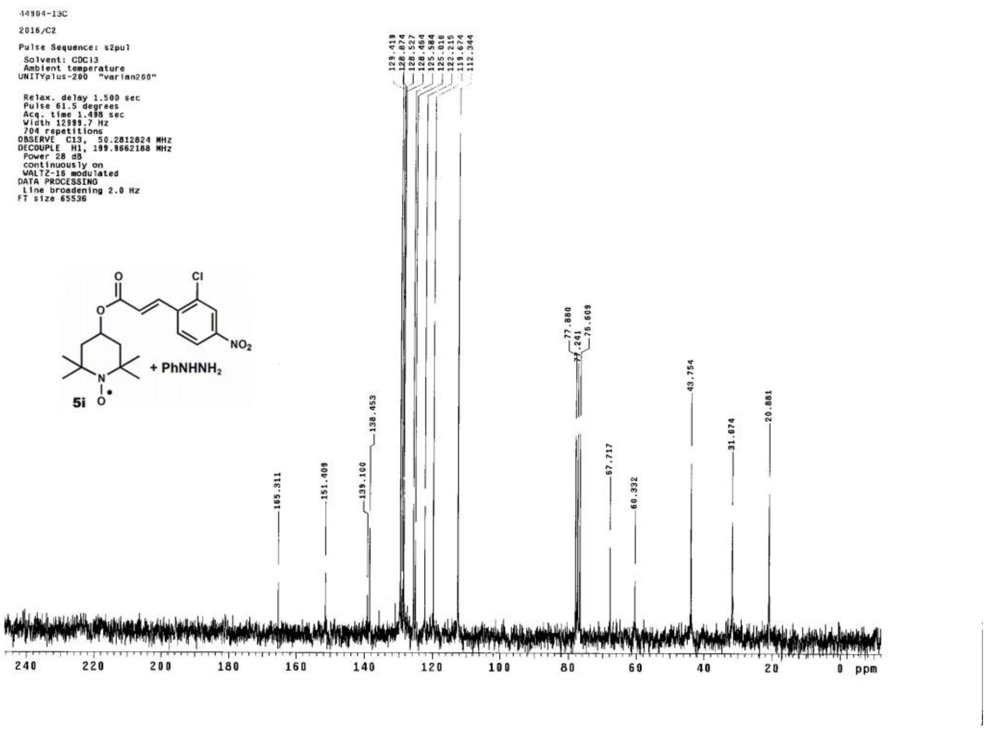

Fig. S42. 2,2,6,6-Tetramethyl-1-oxyl-4-piperidyl 3-*E*-(2-chloro-4-nitrophenyl)acrylate (**5i**) + $C_6H_5NHNH_2$, ^{13}C NMR

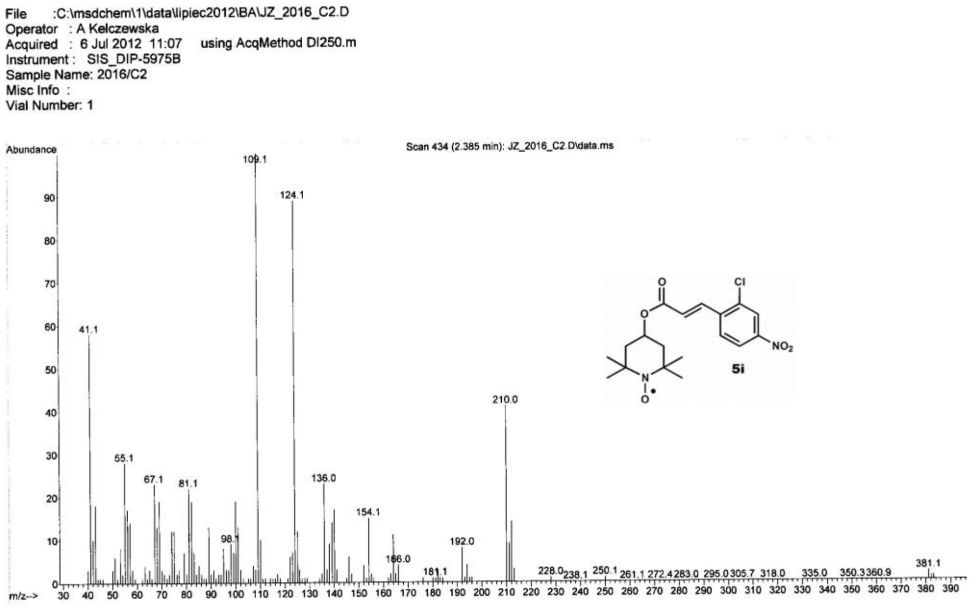

Fig. S43. 2,2,6,6-Tetramethyl-1-oxyl-4-piperidyl 3-*E*-(2-chloro-4-nitrophenyl)acrylate (**5i**), EI MS

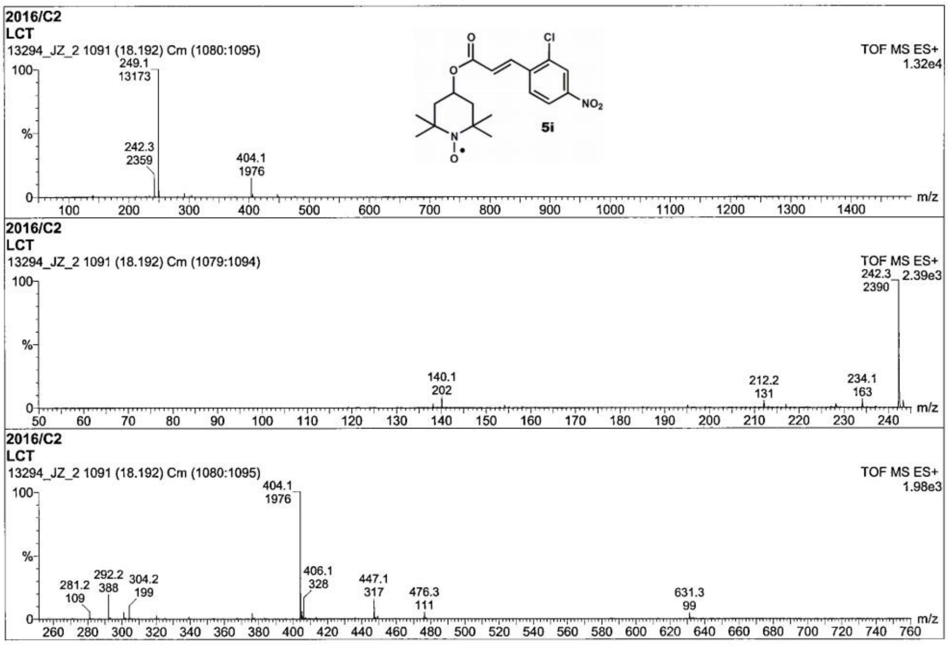

Fig. S44. 2,2,6,6-Tetramethyl-1-oxyl-4-piperidyl 3-*E*-(2-chloro-4-nitrophenyl)acrylate (**5i**), ESI MS

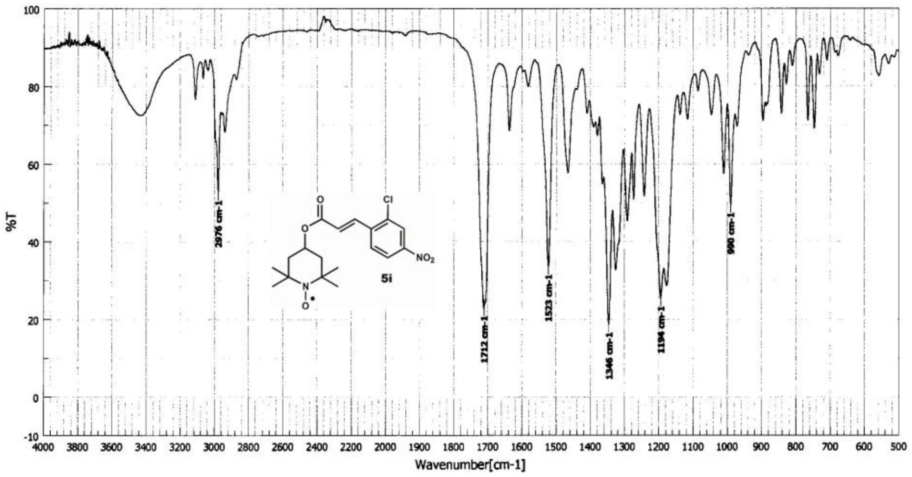

Sample Name	2016/C2 (0,7mg/270mg KBr)
Resolution	1 cm-1
Accumulation	30
Apodization	Cosine
Date/Time	112-07-06 13:36

Fig. S45. 2,2,6,6-Tetramethyl-1-oxyl-4-piperidyl 3-*E*-(2-chloro-4-nitrophenyl)acrylate (**5i**), IR
